# Mesenchymal stem cell-induced metabolic reprogramming of EGFR-wild-type tumor cells drives therapeutic resistance in EGFR-mutant non-small cell lung cancer

**DOI:** 10.1186/s13046-026-03748-w

**Published:** 2026-07-11

**Authors:** Hongyu Bie, Jiarui Li, Jialin Liu, Jian Zhou, Tierun Wang, Xu Guo, Jiawei Liu, Yi You, Hui Huang, Shijia Li, Wanqiao Li, Xiubao Ren, Meng Wang, Wencheng Zhang, Cihui Yan

**Affiliations:** 1 Department of Immunology, Tianjin Medical University Cancer Institute & Hospital, Tianjin, 300060 China; 2 Department of Radiation Oncology, Tianjin Medical University Cancer Institute & Hospital, Tianjin, 300060 China; 3 Department of Lung Cancer, Tianjin Medical University Cancer Institute & Hospital, Tianjin, 300060 China; 4 Tianjin Lung Cancer Center, Tianjin, 300060 China; 5 Tianjin’s Clinical Research Center for Cancer, Tianjin, 300060 China; 6 National Clinical Research Center for Cancer, Tianjin, 300060 China; 7 State Key Laboratory of Druggability Evaluation and Systematic Translational Medicine, Tianjin, 300060 China; 8 Key Laboratory of Cancer Immunology and Biotherapy, Tianjin, 300060 China; 9 Key Laboratory of Cancer Prevention and Therapy, Tianjin, 300060 China

**Keywords:** Mesenchymal stem cell, Epidermal growth factor receptor, Non-small cell lung cancer, Metabolic reprogramming, Tyrosine kinase inhibitor

## Abstract

**Background:**

To explore mesenchymal stem cell (MSC)-driven metabolic reprogramming of EGFR-wild-type (EGFR-wt) tumor cells contributing to tyrosine kinase inhibitor (TKI) resistance in EGFR-mutant (EGFR-mt) non-small cell lung cancer.

**Methods:**

Isolate MSCs from paired tumor and non-tumor lungs of EGFR-mt and EGFR-wt patients. Integrate transcriptional RNA sequencing, targeted free fatty acid and energy metabolomics to characterize signaling pathways promoting MSC–EGFR-wt tumor cell interaction within EGFR-mt tumors. Assess spatial correlation between these cells in TKI-resistant and TKI-sensitive patients using immunohistochemistry and multiplex immunofluorescence.

**Results:**

EGFR-mt lung cancer-derived MSCs (mtLC-MSCs) promoted EGFR-wt tumor proliferation, and reduced TKI efficacy in vivo. Mechanistically, mtLC-MSCs increased fatty acid levels, supporting stem-like features in EGFR-wt tumor cells. These effects were mediated by IL-6 and IL-1α secreted from mtLC-MSCs, which enhanced S100A9 expression in EGFR-wt tumor cells and activated downstream c-Myc/β-catenin-FASN signaling, driving fatty acid synthesis. TKI plus IL-6 pathway blockade reversed resistance in mice. In EGFR-mt NSCLC patients, the proportions mtLC-MSCs was comparable with mtTF-MSCs. TKI-resistant samples exhibited greater mtLC-MSCs wrapping density around tumor masses than sensitive samples. EGFR-mt tumor cells showed closer spatial proximity to mtLC-MSCs than did EGFR-wt cells.

**Conclusion:**

These findings firstly reveal the spatial heterogeneity and metabolic reprograming of EGFR-wt tumor cells driven by mtLC-MSCs in EGFR-mt tumors, providing novel insight into therapeutic resistance and potential combination strategies for TKI-resistant patients.

**Supplementary Information:**

The online version contains supplementary material available at 10.1186/s13046-026-03748-w.

## Introduction

Activating mutations in the epidermal growth factor receptor (EGFR) define a distinct molecular subtype of non-small cell lung cancer (NSCLC), occurring in approximately 10–15% of cases in Western populations and up to 40–50% in East Asian cohorts [1, 2]. The application of EGFR tyrosine kinase inhibitors (TKIs) has markedly improved clinical outcomes for these patients, producing higher response rates and longer survival compared with chemotherapy [3, 4]. However, the emergence of primary and acquired resistance is nearly universal, and disease progression remains the major barrier to durable benefit [5,6]. Revealing the mechanisms that underlie resistance in EGFR-mutant NSCLC is therefore a central challenge in the field.

Although EGFR-mutant (EGFR-mt) tumors are often treated as uniform entities, accumulating evidence indicates that they contain heterogeneous subclonal populations, including tumor cells lacking EGFR mutations [7]. These EGFR-wild-type (EGFR-wt) cells may pre-exist within the tumor or emerge under the selective pressure of TKI therapy. Amplification of EGFR-wt alleles has been reported as a mechanism of acquired resistance to third-generation EGFR TKIs, indicating the functional contribution of wild-type signaling to therapeutic escape [8]. In addition, ligand-driven activation of EGFR-wt cells has been shown to sustain tumor growth in preclinical models [9]. Together, these studies suggest that EGFR-wt tumor cells are not passive bystanders but active participants in resistance. However, the mechanisms that enable their persistence within an EGFR-mt tumor context remain poorly defined. Specifically, whether metabolic reprogramming supports the survival of EGFR-wt tumor cells under TKI pressure has not been addressed.

The tumor microenvironment (TME) is a critical determinant of therapeutic response [10–12]. Mesenchymal stromal cells (MSCs) constitute a stromal progenitor population residing in normal lung tissues and can be reprogrammed into lung cancer-associated MSCs (LC-MSCs) within the TME, particularly in metabolic regulation, remain insufficiently explored [13, 14]. In NSCLC, only limited studies have suggested that MSC-derived signals may enhance cancer cell aggressiveness [15], and the adaptive states of MSCs within EGFR-mt NSCLC are largely unknown [16]. Given the profound metabolic plasticity of EGFR-mt NSCLC, understanding how tumor-educated MSCs affect nearby tumor cells is important. These gaps highlight the need to define the functional roles of MSCs in the EGFR-mt TME.

In the present study, we sought to address this question by integrating multi-omics analyses with validation in patient samples. We demonstrate that EGFR-wt tumor cells engage in reciprocal crosstalk with MSCs derived from EGFR-mt lung cancers (mtLC-MSCs), leading to metabolic reprogramming characterized by enhanced lipogenesis. Mechanically, we identify an IL-6/IL-1α–S100A9-β-catenin/c-Myc signaling pathway that links stromal inflammatory cues to the activation of lipogenic enzymes, thereby promoting stem-like properties and persistence of EGFR-wt clones. By delineating how EGFR-wt tumor cells adapt within the EGFR-mt TME, our findings reveal a previously unrecognized stromal-tumor metabolic interaction that contributes to therapeutic resistance and may offer new avenues for intervention in EGFR-mt NSCLC.

## Methods

### MSC preparation

BMMSCs (bought from the Cell Resource Center, Shanghai Institute of Life Sciences, Chinese Academy of Sciences) were cultured in DF-12 medium supplemented with 10% fetal bovine serum (FBS, 16000044, Gibco, USA) and 2 mM L-glutamine (2523105, Gibco, USA). HUMSCs, lung LC-MSCs and their matched-paired tumor-free MSCs (TF-MSCs) were respectively isolated from the umbilical cords, tumor tissues and paired adjacent non-tumorous lung tissues of three NSCLC patients (one EGFR-mt and two EGFR-wt) using the tissue explant method. Briefly, freshly resected tissues were immediately immersed in cold phosphate-buffered saline (PBS) containing 100 U/mL penicillin and 100 µg/mL streptomycin. The tissues were rinsed with cold PBS to remove blood, meticulously dissected to remove blood vessels, and minced into approximately 8 mm³ pieces. These tissue explants were then plated onto T-75 culture flasks pre-coated with 2 mL of DF-12 medium supplemented with 2 mM L-glutamine and 10% FBS. The flasks were incubated upside down overnight at 37 °C in a humidified 5% CO₂ atmosphere. The following day, the flasks were carefully turned upright. The explants were initially supplemented with 1 mL of fresh medium daily for the first three days, followed by 3 mL on the fourth day. Thereafter, half of the medium was replaced every three days. After approximately two weeks of culture, the outgrowing MSCs were subjected to the first passage. These cells exhibited typical fibroblastic and spindle-shaped morphology and maintained this typical morphology during subsequent passages. Cells were subcultured at a density of 4,000 cells/cm². MSCs were identified by their multiple differentiation capacity and characteristic surface phenotypes as determined flow cytometry. Surface marker profiling confirmed that the cell populations were strongly positive for CD73, CD90, CD166, and CD105, while being negative for hematopoietic and endothelial markers, including CD34, CD45, CD14, CD19, and HLA-DR. MSCs from passages 2 to 10 were used in the following experiments.

### Tumor cell culture and co-culture assays

NSCLC cell lines A549, H1385, H226, H1975 and PC9 (bought from the Cell Resource Center, Shanghai Institute of Life Sciences, Chinese Academy of Sciences) were cultured in RPMI-1640 medium supplemented with 10% FBS, 2 mM L-glutamine, 100 U/mL penicillin, and 100 µg/mL streptomycin. In order to in vivo live imaging and cell sorting, A549 cell line was modified to stably express luciferase and enhanced green fluorescent protein (eGFP) using lentiviral transduction (details in Supplementary materials). For indirect co-culture, a Transwell system (0.4 μm pore size) was used to prevent direct cell–cell contact while allowing the exchange of soluble factors in the 6-well plates. MSCs (2 × 10⁵ cells) were seeded in the upper chamber, while A549 and H1385 cells (2 × 10⁵ cells each) were seeded in the lower chamber. Cells were maintained in complete culture medium and co-cultured for 72 h. For direct co-culture, A549 and H1385 cells (2 × 10⁵ cells each) were co-seeded with MSCs (2 × 10⁵ cells) in 6-well plate to allow direct cell–cell contact. Cells were cultured in 1640 medium containing 10% FBS for 72 h. eGFP-labeled tumor cells were flow-sorted prior to downstream analyses.

### Conditioned medium preparation and treatment

Conditioned medium preparation: MSCs in good growth condition were seeded at a density of 5 × 10^5^ cells per T-75 flask and cultured with 8 mL of DF-12 medium supplemented with 10% FBS. After 72 h, the culture medium was collected and centrifuged at 3000 rpm for 5 minutes at 4℃. The supernatant was subsequently filtered through a 0.22-µm membrane to remove cellular debris. The processed medium was aliquoted and stored at -80 °C for subsequent use. Cell Treatment: tumor cells in good growth condition were digested and resuspended. The cells were then cultured in a 1:1 (v/v) mixture of the prepared conditioned medium and fresh 1640 medium containing 10% FBS.

### Antibodies

Antibodies used for Western blot: S100A9 (CST, 72590, 1:1000), FASN (CST, 3180, 1:1000), c-Myc (CST, 5605, 1:1000), β-catenin (abcam, ab32572, 1:5000), GLUT1 (CST, 12939, 1:1000), β-actin (CST, 4970, 1:1000), Oct-4 (abcam, ab109183, 1:2000), CD44 (abcam, ab51037, 1:5000), SOX2 (CST, 14962, 1:1000), NF-κB p65 (HUABIO, ET1603-12, 1:5000), Phospho-NF-κB p65 (S536) (HUABIO, HA723223, 1:5000), Phospho-Stat3 (CST, 9145, 1:1000), Stat3 (CST, 9139, 1:1000), Phospho-p44/42 MAPK(Erk1/2) (Thr202/Tyr204) (CST, 4377, 1:1000), p44/42 MAPK (Erk1/2) (CST, 9102, 1:1000), Goat anti-Mouse IgG-HRP Antibody (absin, abs20039,1:5000), and Goat anti-Rabbit IgG-HRP Antibody (absin, abs20040, 1:5000). Antibodies used for Immunofluorescence: β-catenin (abcam, ab32572, 1:250), c-Myc (CST, 5605, 1:400), S100A9 (CST, 72590, 1:400), Goat anti-Rabbit IgG (H + L) Highly Cross-Adsorbed Secondary Antibody (thermo, A-11034,1:200). Antibodies used for immunohistochemistry (IHC) and multiplex immunofluorescence (mIF): SOX2 (CST, 14962, 1:300), CD44 (abcam, ab51037, 1:50), Oct-4 (abcam, ab109183, 1:5000), CD133 (abcam, ab222782, 1:500), β-catenin (abcam, ab32572, 1:250), CD105 (abcam, ab170943, 1:250), EGFR (L858R) (CST, 3197, 1:100), EGFR (E746-A750del) CST, 2085, 1:100), S100A9 (CST, 34425, 1:400), FASN (CST, 3180, 1:50), CK (GT, GM35154, RTU), IL-6 (abcam, ab290735,1:500), and IL-1α (abcam, ab300501, 1:5000).

### SiRNA transfection

Transient transfection of siRNA or plasmids was performed using Lipofectamine 3000 transfection reagent, following the manufacturer’s protocol. Tumor cells were first treated with conditioned medium for 24 h, followed by siRNA transfection for 8 h, and subsequently cultured in normal medium for 48 h. SiRNA oligonucleotides targeting S100A9, c-Myc, β-catenin, and FASN, along with a control siRNA and a β-catenin overexpression plasmid, were all purchased from Tsingke Biotechnology (Beijing, China). The knockdown efficiency was assessed using real-time PCR and Western blot analysis, following which the two siRNAs demonstrating the highest knockdown efficacy were selected for further studies. Their sequences are listed in Supplementary Table S1.

### Real-time quantitative PCR

Total RNA was extracted from cells using a commercial RNA extraction kit (Takara, 9767, Japan). RNA concentration was quantified using a NanoDrop spectrophotometer (260/A280). An equal amount of total RNA (1 µg) was used for each reverse transcription reaction using the PrimeScript RT Reagent Kit (RR036A, Takara, Japan) in a final volume of 20 µL. The resulting cDNA product was diluted 10-fold with nuclease-free water, and 2 µL of the diluted cDNA was used per qPCR reaction. Quantitative real-time PCR was performed using qPCR SYBR Green Master Mix (A25742, Thermo, USA) on an ABI 7500 real-time PCR system. The relative expression levels of target genes were normalized and calculated using the 2–ΔΔCt method. β-actin gene expression was used for normalization of each sample. All primer sequences were synthesized by Tsingke Biotech and are listed in Supplementary Table S2.

### Western blot

Cells were lysed on ice for 30 min using RIPA lysis buffer supplemented with protease and phosphatase inhibitors. The lysates were then centrifuged at 12,000 rpm for 10 min at 4 °C to collect the supernatant. Protein concentration was determined using a bicinchoninic acid (BCA) assay kit (23227, Thermo, USA) following the manufacturer’s instructions. Each lysate was diluted to a final concentration of 3 µg/µL. And equal amounts of proteins (30 µg per lane) were mixed with loading buffer, denatured by boiling at 100 °C for 5 min, and separated by SDS-PAGE. Subsequently, the proteins were transferred onto polyvinylidene difluoride (PVDF) membranes. After blocking with 5% skim milk in TBST for 1 h at room temperature, the membranes were incubated with specific primary antibodies overnight at 4 °C. Following three washes with TBST, the membranes were incubated with horseradish peroxidase (HRP)-conjugated secondary antibodies for 1 h at room temperature. After final washes, immunoreactive bands were visualized by enhanced chemiluminescence (ECL) substrate and captured using a chemiluminescence imaging system.

### Flow Cytometry

Surface markers of MSCs from multiple sources were assessed using flow cytometry. MSCs were harvested by enzymatic digestion when reached 80% confluence. These cells were then resuspended in staining buffer and aliquoted at 100 µL (10^7^/mL) in tubes. Each aliquot was stained with fluorochrome-conjugated mouse anti-human antibodies against CD73, CD90, CD105, CD166, CD14, CD19, CD34, CD45, and HLA-DR, and incubated for 30 min on ice. Following incubation, the cells were washed twice, resuspended in staining buffer, and immediately analyzed using a flow cytometer (CytoFLEX LX, Beckman, USA). The flow cytometry antibodies used are listed in Supplementary Table S3.

For sorting eGFP-positive tumor cells from allogeneic tumors, freshly collected tumor tissues from sacrificed mice were firstly cut into ~ 3 mm^3^ pieces, placed in Miltenyi disassociation solution (Miltenyi Biotec, 130-095-929, Germany), and digested using the Miltenyi gentleMACS™ Octo Dissociator. The single-cell suspensions were filtered through a 70-µm cell strainer to remove aggregates. Purified eGFP-positive tumor cells were sorted from the mixed cell population by flow cytometry based on their eGFP fluorescence (FACSAria III, BD, USA). Flow cytometry was also applied to sort eGFP-positive tumor cells from direct co-cultures.

### Immunohistochemistry

Paraffin-embedded tumor specimens were cut into 4 μm sections. After deparaffinization and rehydration, antigen retrieval was performed using either pH 6.0 citrate buffer or pH 9.0 EDTA. Sections were blocked with 1% bovine serum albumin (BSA) and incubated with the primary antibody overnight at 4 °C. On the following day, sections were incubated with an HRP-conjugated secondary antibody for 1 h at room temperature. Signal was developed using a streptavidin-peroxidase system with diaminobenzidine (DAB), and nuclei were counterstained with hematoxylin. Sections were then dehydrated, cleared, and mounted for microscopic evaluation. IHC images were acquired using a digital slide scanner (KF-PRO-040-HI, KFBIO, China). Positive expression was assessed using the H-score, which was determined by multiplying staining intensity by the percentage of positive cell. Four serial sections were used to identify EGFR mutation (exon 19 deletion [19-del] or L858R point mutation [L858R]), FASN, S100A9, and SOX2 expression in tumor cells. The specificity of EGFR mutation-specific antibodies was validated using both positive controls (cell lines with known mutations: PC9 for exon 19 deletion, and H1975 for L858R point mutation) and negative controls (EGFR-wide-type cells lines A549 and paired normal lung epithelium section) (Supplementary Fig S1). All patients included had undergone clinical genotyping (NGS or qPCR).

### Multiplex immunofluorescence

Three staining panels were established in mIF staining. Panel 1 (IL-1α-520/IL-6-620/CD105-690) to designed to identify IL-6 and Il-1α expression in mtLCMSC (*n* = 3 patients). Using paired tumor and normal lung tissue from 23 patients (*n* = 23 pairs), panel 2 (CD105-690/EGFR-620/CK-780) was used to compare the abundance of MSCs in NSCLC tumor and normal lung tissues, and to independently validate, specifically within the tumor, the spatial distribution patterns of mtLC-MSCs relative to tumor cells. Panel 3 (EGFR [19-del or L858R]-620/FASN-520/CD105-690/S100A9-570/CK-780) to set up to investigate the spatial interaction between the subsets of mtLC-MSCs and EGFR-wt tumor cells within tumor tissues (*n* = 5 patients). Staining was performed using the Multiplex Immunohistochemistry Kit Seven Color TSA-RM-827,298 (10268100100, Panovue Biotechnology, China). A five-cycle staining procedure was carried out according to the manufacturer’s instructions. Primary antibodies were incubated for one hour at room temperature. Cell nuclei were visualized with 4′,6-diamidino-2-phenylindole (DAPI). For multispectral imaging, whole slides were scanned at×20 in rapid fluorescence mode using a Polaris digital slide scanner (Vectra Polaris, Akoya Biosciences, USA).

### Imaging and spatial analysis

HALO software (Indica Labs; Version 3.6.4134, RRID: SCR_018350) was used to analyze each whole-slide image. For serial IHC images analyses, tumor cells were firstly segmented and identified at the single-cell level based on cytologic and architectural morphology on the EGFR-stained slides. After color deconvolution and optical-density measurements, tumor cells were classified using predefined thresholds as EGFR-negative (EGFR-wt) or EGFR-positive (EGFR-mt). Using spatial coordinates, all EGFR-wt cells and all EGFR-mt cells were each unified to generate two tumor-region masks. HALO’s serial-section registration and coordinate-mapping tools, combining rigid (translation/rotation/scale) and local elastic alignment, were then used to propagate these regions from the EGFR slide to adjacent serial sections (FASN, S100A9, SOX2) for co-localization across sections. Marker expression of FASN, S100A9, and SOX2 was subsequently quantified within the EGFR-wt and EGFR-mt regions.

For mIF staining analyses, HE-stained slides were firstly used to excluded normal epithelial cells, since pan-Cytokeratin (CK) expression alone could not distinguish them from tumor cells. The remaining CK+EGFR+ cells were defined as EGFR-mt tumor cells, while CK+EGFR- cells were classified as EGFR-wt tumor cells. MSCs were identified as CD105-positive cells with elongated and spindle-shaped morphology. For spatial analysis, nearest-neighbor distances were calculated from core cells to other subsets, with each core cell assigned a single nearest neighbor. The mean nearest-neighbor distance was determined as the average of all nearest-neighbor values across the slides. To calculated mean closest distance within 150 μm, distance greater than 150 μm was excluded prior to averaging.

### Chromatin immunoprecipitation

Chromatin immunoprecipitation (ChIP) assays were performed using an enzymatic ChIP kit (26157, Thermo, USA) according to the manufacturer’s protocol. In brief, 5 × 10^7^cells were fixed with formaldehyde to cross-link proteins to DNA. The chromatin was then fragmented using micrococcal nuclease. Sheared chromatin was immunoprecipitated overnight at 4 °C with specific antibodies against the proteins of interest. Normal IgG was included as a negative control. Following immunoprecipitation, the protein-DNA complexes were eluted and cross-links were reversed. The purified DNA was subsequently analyzed by real-time quantitative PCR to examine the enrichment of specific genomic regions. The primers used in this study are listed in Supplementary Table S4.

### FFA assay

FFA levels in both tumor cell extract and liquid samples were quantified using a commercial colorimetric assay kit (Abcam, ab65341, USA) per the manufacturer’s protocol. Briefly, tumor cells were extracted with chloroform containing 1% Triton X-100, and the resulting organic phase was dried and reconstituted in assay buffer. Liquid samples were tested directly. Subsequently, all samples were incubated with acyl-CoA synthetase and an enzyme mix, and the absorbance of the reaction product was measured at 570 nm. FFA concentrations were determined against a palmitic acid standard curve and normalized for sample volume and dilution factors.

### Mice xenograft experiment

A subcutaneous xenograft tumor model was established in female BALB/c nude mice aged 5 weeks (Beijing Vital River Laboratory Animal Technology, Beijing, China). To investigate EGFR-wt tumor growth in the presence of mtLC-MSCs, mice were randomly divided into three groups as follows: (1) 5 × 10⁵ A549 cells (control group); (2) 5 × 10⁵ A549 cells with 2 × 10⁵ mtTF-MSCs (mtTF-MSC group); and (3) 5 × 10⁵ A549 cells with 2 × 10⁵ mtLC-MSCs (mtLC-MSC group). Injection of 5 × 10⁵ A549 cells represented the minimal cell number required for tumor formation. To evaluate the impact of EGFR-wt tumor cells on TKI treatment efficacy, mice were inoculated with 9 × 10⁵ PC9 cells and 3 × 10⁵ A549 cells, or co-implanted with 3 × 10⁵ mtLC-MSCs or mtTF-MSCs, respectively. When the tumor volume reached 50–100 mm³, mice were randomly assigned into treatment and control groups (*n* = 5 per group). The treatment group received daily intraperitoneal injections of TKI osimertinib (10 mg/kg). To explore treatment outcome of osimertinib combined with IL-6 blockade, mice were inoculated with 9 × 10⁵ PC9 cells and 3 × 10⁵ A549 cells, along with 3 × 10⁵ mtLC-MSCs. When tumor attained 50–100 mm³ in volume, the mice were randomly assigned into the following treatment groups (*n* = 5 per group): osimertinib monotherapy, Tocilizumab monotherapy, and combination therapy. Osimertinib was given as described above. Tocilizumab was administered at a dose of 5 mg/kg via intraperitoneal injection every other day.

Because A549 cells were stably expressed firefly luciferase, their growth in the co-implanted models was monitored in vivo using the IVIS^®^ Imaging System (Xenogen 100, Caliper Life Sciences, USA). For bioluminescence imaging, D-luciferin (APExBIO, C3654, USA) was administered intraperitoneally at 150 mg/kg, and imaging was performed 10 min post-injection. For total tumor growth, tumor length (L) and width (W) were measured every other day using a caliper for a duration of 20 days. Tumor volume (V) was calculated as V = (L × W²) / 2. At the experimental endpoint, tumors were excised from sacrificed mice for further analysis. To quantify the proportion of A549 and PC9 cells directly in tumor tissue, paraffin-embedded sections were prepared. A549 cells were visualized by eGFP fluorescence on unstained sections. PC9 cells were identified by EGFR 19-del immunohistochemistry on adjacent sections. The proportion of each cell type among all nucleated cells was counted across the entire section. All animal experiments were approved by the Laboratory Animal Ethics Committee of Tianjin Medical University Cancer Institute & Hospital (Ethical number: AE-2022040).

### Statistical analysis

Statistical analyses were conducted using GraphPad Prism version 9.0. Data are presented as the mean ± SD or mean ± SEM, as indicated in the figure legends. To compare growth curves, we used the two-way ANOVA with Dunnett’s multiple comparisons test. When comparing more than two groups, we performed one-way ANOVA. For comparisons between two groups, the Student’s t-test, Wilcoxon signed-rank test or the nonparametric Mann–Whitney U test was employed, depending on data distribution. For pathway enrichment analyses and in vivo experiments involving multiple groups, FDR-adjusted q-values were calculated using Benjamini-Hochberg method. For GSEA, six predefined gene sets were included, and normalized enrichment scores (NES), nominal p-values, and FDR-adjusted q-values were reported. For cell-based experiment, each analysis involved a single pre-specified comparison versus the designated control group (mtLC-MSC group). No pairwise multiple testing was performed across treatment groups; therefore, multiple-testing correction was not applicable. A value of *P* < 0.05 or FDR q < 0.05 indicated statistical significance. Significant differences among groups are indicated as *P < 0.05, **P < 0.01, ***P < 0.001, **** *P* < 0.0001.

Other methods, including Lentivirus transfection, cell proliferation assay, activation and functional evaluation in T cells, cellular immunofluorescence, ELISA, metabolite assays, targeted metabolomics, and transcriptomics are described in detail in the Supplementary Methods.

## Results

### Enhanced IL-6 and IL-1α expression distinguishes mtLC-MSCs from wtLC-MSCs and TF-MSCs

**Fig. 1:**
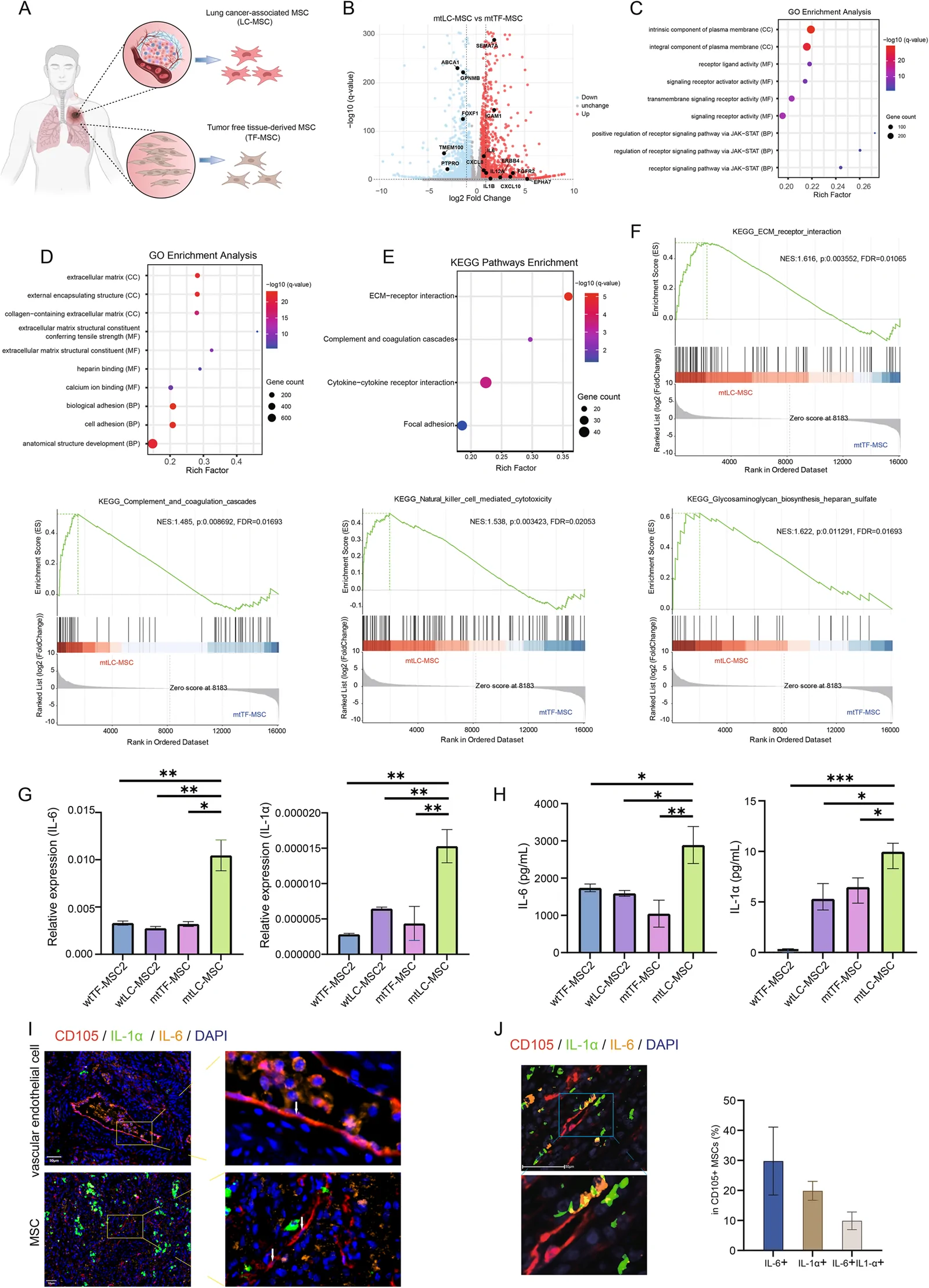
mtLC-MSCs express higher levels of IL-6 and IL-1α compared with wtLC-MSC and TF-MSC. **A** Schematic diagram isolating LC-MSCs and TF-MSCs from NSCLC and paired non-cancer lung tissues, respectively. **B** Differentially expressed genes between mtLC-MSCs and mtTF-MSCs identified by RNA sequencing. **C**, **D** GO enrichment analysis revealed significant enrichment in membrane components, receptor activity and signaling pathways in mtLC-MSCs. **E** KEGG enrichment analysis indicated enriched inflammation/microenvironment-related pathways in mtLC-MSC. **F** GSEA of immune and matrix pathways in mtLC-MSC vs. mtTF-MSC. **G** mtLC-MSCs exhibited elevated transcriptional levels of IL-6 and IL-1α. **H** ELISA assays demonstrated elevated levels of IL-6 and IL-1α in mtLC-MSCs. **I**, **J** Multiple immunofluorescence assessed IL-6 and IL-1α expression within EGFR-mt NSCLC tissues. mtLC-MSCs and vascular endothelial cells both expressed CD105, but presented different morphology (**I**); mtLC-MSCs expressed IL-6 and IL-1α (**J**). Statistical results were shown as mean ± SD. *P<0.05, **P<0.01, ***P<0.001, **** P < 0.0001. One-way ANOVA test. Q-values adjusted by FDR for multiple group comparisons. mt/wtLC-MSC, EGFR mutation/wild-type lung cancer mesenchymal stromal cell; mt/wtTF-MSC, EGFR mutation/wild type tumor-free mesenchymal stromal cell; NSCLC, non-small cell lung cancer; SD, standard deviation

To investigate the characteristics of MSCs educated by the TME of EGFR-mt NSCLC, we firstly isolated LC-MSCs from one patient with an EGFR-mt tumor and from two patients with EGFR-wt tumors (Fig. 1A; Supplementary Table S5). In parallel, we obtained matched TF-MSCs from the adjacent non-tumorous lung tissues of these patients. We defined LC-MSC and TF-MSCs from the EGFR-mt tumor as mtLC-MSCs and mtTF-MSCs, and those from the EGFR-wt tumors as wtLC-MSCs and wtTF-MSCs. These MSCs exhibited multipotent differentiation capacity into adipocytes, osteocytes and chondrocytes, and displayed a characteristic immunophenotypes, being positive for CD73, CD90, CD166 and CD105, while negative for CD14, CD19, CD34, CD45 and HLA-DR, as previously described (Supplementary Fig S2). 

Comparative transcriptomic analysis between mtLC-MSCs and mtTF-MSCs revealed that 2478 differentially expressed genes (DEGs), including 1017 upregulated and 1461 downregulated genes in mtLC-MSCs compared with mtTF-MSCs (Fig. 1B; Supplementary Table S6). Specially, the inflammatory factors IL-6 and IL-1β, together with the chemokines CXCL10 and CXCL8, were highly expressed in mtLC-MSCs (Fig. 1B). Gene Ontology (GO) pathway analysis based on the DEGs revealed enrichment in component of membrane signaling, receptor activity, JAK-STAT signaling pathway (Fig.1C) and extracellular matrix (ECM) remodeling and adhesion (Fig. 1D). KEGG pathway analysis identified similar enriched pathways, including ECM-receptor interaction, cοmplement and coagulation cascades, cytokine-cytokine receptor, focal adhesion (Fig. 1E; Supplementary Table S7). Gene set enrichment analysis (GSEA) revealed that the complement and coagulation cascades, natural killer cell mediated cytotoxicity, glycosaminoglycan biosynthesis heparan sulfate, ECM receptor interaction, cytokine-cytokine receptor, and focal adhesion were upregulated in mtLC-MSCs (Fig. 1F; Supplementary Table S8). These results suggested that the mtMSC-derived inflammatory factors induce inflammatory signaling pathways and ECM remodeling, which might contribute to the altered TME in EGFR-mt NSCLC.

We then assessed IL-6 and IL-1β, along with IL-1α, another cytokine that shares the IL-1R1 receptor with IL-1β, across multiple MSC populations. The mtLC-MSCs exhibited the highest levels of IL-6 and IL-1α at both transcriptional and secretion protein levels, compared with mtTF-MSCs, wtLC-MSCs, or wtTF-MSCs (Fig. 1G, H). Since IL-1β secretion was too low to be detected by transcriptional and ELISA, it was not included in further analyses. In addition, IL-6 and IL-1α expression was assessed in EGFR-mt tumor tissues by multiple immunofluorescent staining. Although both MSCs and endothelial cells exhibited CD105, they displayed distinct morphologies: MSCs exhibited an elongated, spindle-shaped appearance, whereas endothelial cells appeared polygonal and flattened, forming a continuous monolayer (Fig. 1I). Combining CD105 expression with characteristic morphology of MSC allows accurate identification of this cell population in tissues. Quantitatively, 29.8% and 19.9% of mtMSCs expressed IL-6 and IL-1α, respectively, and 9.9% of mtMSCs co-expressed both cytokines (Fig. 1J). While tumor cells expressed IL-6 and IL-1α, the transcriptional IL-6 was markedly lower in tumor cells than that in mtMSCs, and highest IL-1α level was observed in EGFR-mt H1975 cells (Supplementary Fig S3). Together, these findings highlight the pro-inflammatory phenotype of mtLC-MSCs and their close interaction with the TME. 

### mtLC-MSCs reprogram EGFR-wt tumor cells toward lipogenesis

**Fig. 2:**
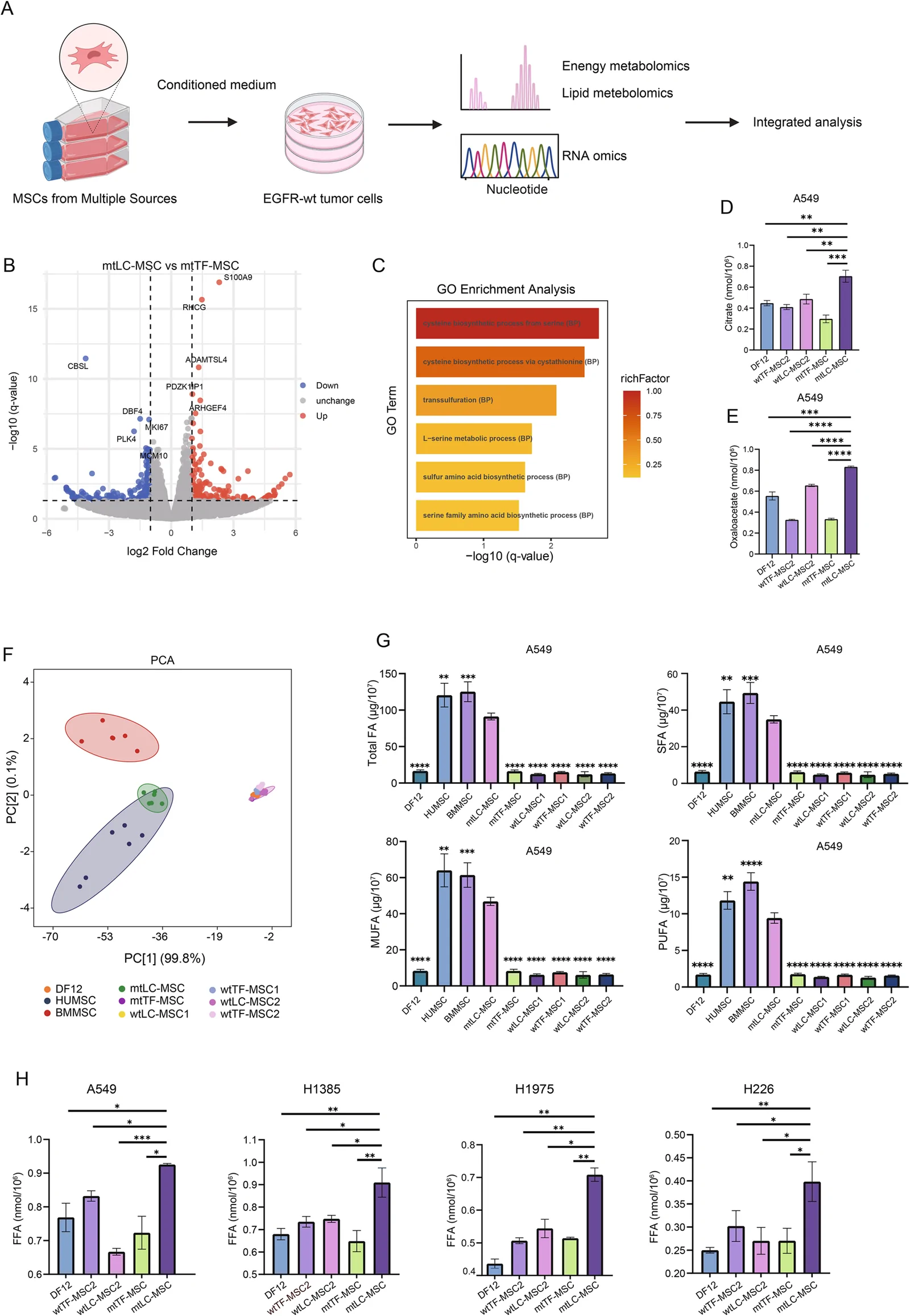
mtLC-MSCs reprogram the metabolism of EGFR-wild-type tumor cells. **A** Strategy for multi-omics characterization of A549 cells exposed to MSC-conditioned medium. **B** Differentially expressed genes in A549 cells treated with mtLC-MSC vs. mtTF-MSC-conditioned medium, identified by RNA sequencing. **C** GO enrichment analysis indicated significant associations with metabolic processes and inflammatory response. **D**, **E** Elevated citrate (**D**) and oxaloacetate (**E**) in A549 cells exposed to conditioned medium. **F**, **G** Targeted metabolomics quantified medium- and long-free fatty acids (FFAs) in A549 cells treated with conditioned medium. **F** Principal components analysis (PCA); **G** FFA levels under different conditioned medium treatments. **H** Highest intracellular FFA levels detected in EGFR-wt tumor cells treated with mtLC-MSC-conditioned medium. exhibited significantly elevated levels of total FA, SFA, MUFA and PUFA. All cells were treated with conditioned medium for 72 h. Statistical results were shown as mean ± SD. *P < 0.05, **P < 0.01, ***P < 0.001, **** P < 0.0001. One-way ANOVA test. Q-values adjusted by FDR for multiple group comparisons. SFA, MUFA, and PUFA denote saturated, monounsaturated, and polyunsaturated fatty acid, respectively

Given in EGFR-mt tumor tissues, mtLC-MSCs likely interact not only with EGFR-mt tumor cells, but also with EGFR-wt tumor cells besides, particularly after TKI-induced death of EGFR-mt cells, we therefore focused on the effects of mtLC-MSC on EGFR-wt tumor cells. To this end, we integrated transcriptomic, lipid metabolomic, and energy metabolomic analyses of EGFR-wt A549 cells treated with conditioned medium from different MSC populations (Fig. 2A). RNA-seq analysis revealed that exposure to mtLC-MSC-conditioned medium resulted in 43 DEGs, with 21 upregulated and 22 downregulated compared with the paired mtTF-MSC derived conditioned medium (Fig. 2B; Supplementary Table S9). Among the upregulated DEGs, S100A9 showed the most significant increase (Fig. 2B). Pathway enrichment analysis indicated that the DEGs were pathway associated with serine and sulfur amino acid metabolism and inflammatory response (Fig. 2C). Consistent with these transcriptional changes, metabolomic profiling demonstrated increased intracellular oxaloacetate and citrate, which are key intermediates of the citrate shuttle that provide cytosolic acetyl-CoA and oxaloacetate for NADPH generation in EGFR-wt A549, H1385, and H226 cells treated with mtLC-MSC-conditioned medium (Fig. 2D and E; Supplementary Fig S4A, B and S5A–C). Functionally, compared with A549 cells treated with mtTF-MSCs- or wt-MSCs-conditioned medium, those exposed to mtLC-MSC-conditioned medium displayed a medium- and long-chain free fatty acid (FFA) profile more similar to cells treated with human umbilical MSCs (HUMSCs)- and bone marrow-derived MSCs (BMMSCs)-conditioned medium (Fig. 2F). Notably, intracellular levels of saturated fatty acid (FA), monounsaturated FA, and polyunsaturated FA were markedly increased in these A549 cells (Fig. 2G; Supplementary Fig S5D, E), a finding further confirmed in EGFR-wt H1385, H1975, and H226 cells (Fig. 2H). These results suggest that mtLC-MSCs were associated with metabolic changes in EGFR-wt tumor cells, consistent with enhanced FA synthesis.

### IL-6 and IL-1α from mtLC-MSCs induces S100A9 and drive lipogenesis via TLR/RAGE in EGFR-wt tumor cells

**Fig. 3:**
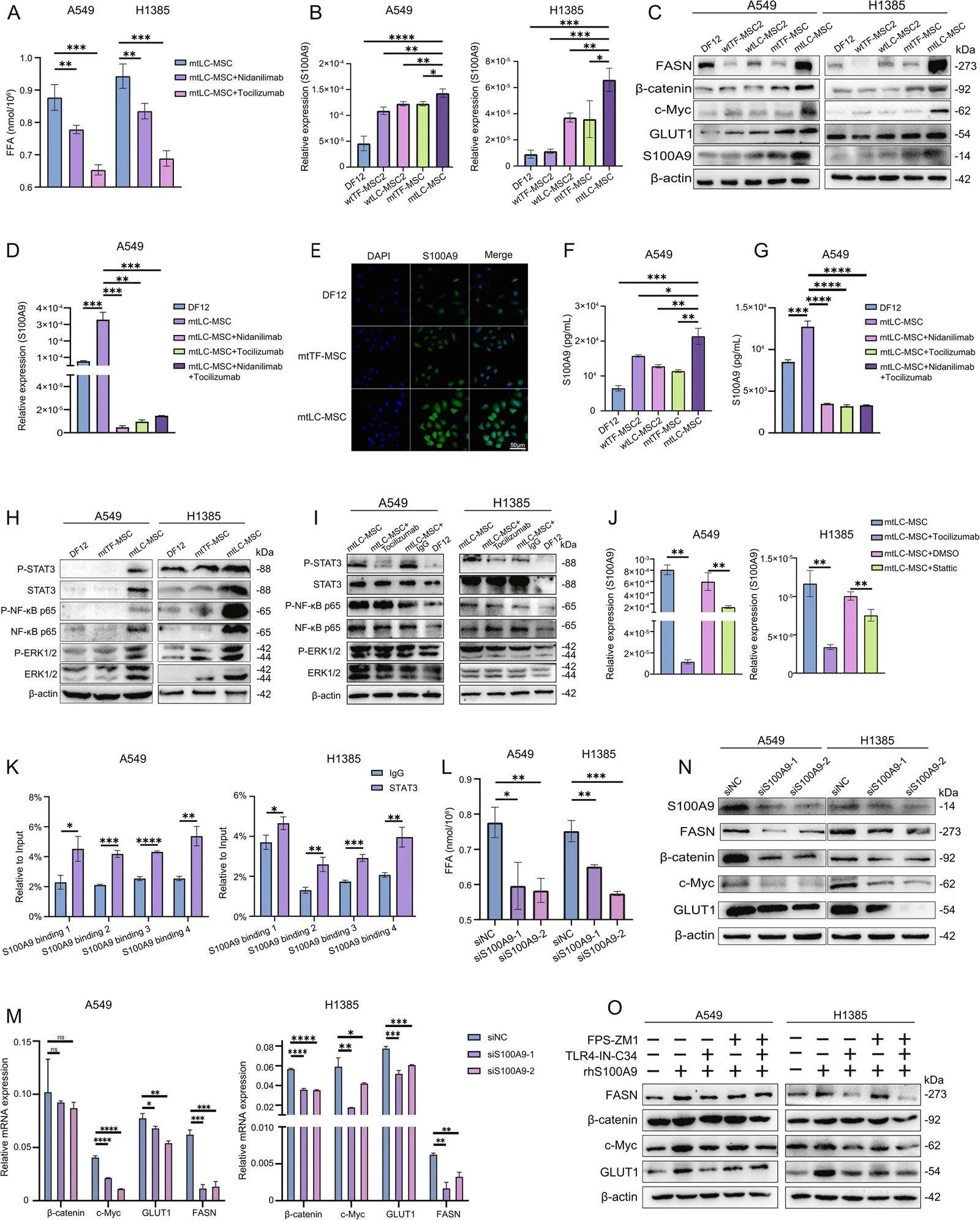
S100A9 promotes the accumulation of fatty acids in EGFR-wild-type tumor cells. **A** Nimotuzumab and Tocilizumab reduced the elevated intracellular FFA levels induced by mtLC-MSCs. **B** Transcriptional S100A9 regulated by conditioned medium. **C** Indicated protein levels under conditioned medium treatment. **D** mtLC-MSC-induced upregulation of S100A9 transcription was reduced by nimotuzumab and tocilizumab. **E** S100A9 accumulated in nucleus after mtLC-MSC-conditioned medium treatment. **F**, **G** S100A9 secretion with conditioned medium (**F**) and blockaded by nimotuzumab and tocilizumab (**G**). **H**, **I** Downstream pathways activated by conditioned medium treatment (**H**) and inhibited by nimotuzumab and tocilizumab (**I**). **J** STAT3 inhibitor stattic decreased mtLC-MSC-induced S100A9 transcription. **K** ChIP-qPCR confirmed STAT3 binding sites in the S100A9 promoter. **L** S100A9 silencing attenuated intracellular FFA levels. **M**, **N** S100A9 silencing reduced transcriptional (**M**) and protein (**N**) levels of downstream targets. **O** Blockade of S100A9 receptors TLR4 and RAGE attenuated protein levels of downstream targets. Conditioned medium treatment was applied for 72 h (**A**–**F**), 30 min (**H**, **I**), or 24 h followed by normal medium for silencing (**L**-**N**). Blocking antibodies and inhibitors were pre-treated for 1 h. Nimotuzumab (20 µg/mL), Tocilizumab (100 ng/mL), Stattic (10 µM), rhS100A9 (500 ng/mL), FPS-ZM1 (10 µM), and TLR4-IN-C34 (10 µM). Statistical results were shown as mean ± SD. *P < 0.05, **P < 0.01, ***P < 0.001, **** P < 0.0001. One-way ANOVA test except panel J and K (Student’s t-test). FFA, free fatty acid; GLUT1, glucose transporters 1; FASN, fatty acid synthase; TLR4, toll-like receptor 4; RAGE, receptor for advanced glycation end products

Building on pathway enrichment of inflammatory responses in EGFR-wt tumor cells exposed to mtLC-MSCs-conditioned medium (Fig. 2C), we investigated whether mtLC-MSCs-derived inflammatory cytokines promote lipogenic activation. In A549 and H1385 cells treated with mtLC-MSCs-conditioned medium, intracellular FFA levels were significantly reduced by blocking IL-6 or IL-1α signaling, with tocilizumab or nidanilimab, (CAN04; IL-1RAP antagonist), respectively; more pronounced decrease observed upon IL-6 inhibition compared with IL-1α blockade (Fig. 3A). Concordantly, mtLC-MSCs-induced increase of S100A9, observed both at mRNA and protein levels (Fig. 3B, C), was markedly diminished at the mRNA level upon IL-6 or IL-1α blockade (Fig. 3D). Under mtLC-MSCs-conditioned medium treatment, S100A9 did not accumulate in nucleus by immunofluorescence (Fig. 3E), but increased secretion into the culture supernatant (Fig. 3F), which was reduced by IL-6 or IL-1α blockade (Fig. 3G). JAK/STAT3 signaling is the canonical downstream pathway of IL-6, whereas NF-κB and MAPK/ERK pathways are shared between IL-6 and IL-1α [17–19]. To determine which pathways mediated IL-6- and IL-1α-induced upregulation of S100A9, we examined phosphorylation of these signaling cascades following treatment with mtLC-MSCs-conditioned medium. The phosphorylated levels of STAT3, p65 and ERK were increased in both A549 and H1385 cells (Fig. 3H). Tocilizumab specifically reversed the enhanced STAT3 phosphorylation, with no impact on p65 and ERK1/2 phosphorylation (Fig. 3I). The STAT3 inhibitor Stattic partially decreased S100A9 transcription compared with tocilizumab (Fig. 3J), and Chip-qPCR assay confirmed STAT3 binding sites in the S100A9 promoter (Fig. 3K). These findings indicate that mtLC-MSC-derived IL-6 and IL-1α upregulate S100A9 in EGFR-wt tumor cells through JAK/STAT3, NF-κB and MAPK/ERK pathways. 

Furthermore, S100A9 knockdown reduced the elevated intracellular FFA levels induced by mtLC-MSCs-conditioned medium (Fig. 3L). Downstream effectors of S100A9, including c-Myc, β-catenin, and the important metabolic associated molecules, FA synthase (FASN), and glucose transporter 1 (GLUT1, also known as SLC2A1), which were upregulated by mtLC-MSCs-conditioned medium or recombinant human S100A9 (rhS100A9) (Fig. 3C; Supplementary Fig S6A, B), were decreased at transcriptional and protein levels when S100A9 expression was inhibited (Fig. 3M, N). As a secreted ligand, S100A9 engages Toll-like receptor 4 (TLR4) and receptor for advanced glycation end products (RAGE) (Supplementary Fig S7) [20, 21]. Pharmacologic inhibition with TLR4-IN-C34 (TLR4) and FPS-ZM1 (RAGE) attenuated S100A9-driven upregulation of c-Myc, β-catenin, FASN, and GLUT1 at both mRNA and protein levels (Supplementary Fig S8; Fig. 3O). These results confirm that the S100A9-TLR4/RAGE axis mediated the lipogenic and metabolic transcriptional program induced by mtLC-MSCs.

### c-Myc and β-catenin mediated S100A9-driven lipogenesis and stemness in EGFR-wt tumor cells

**Fig. 4:**
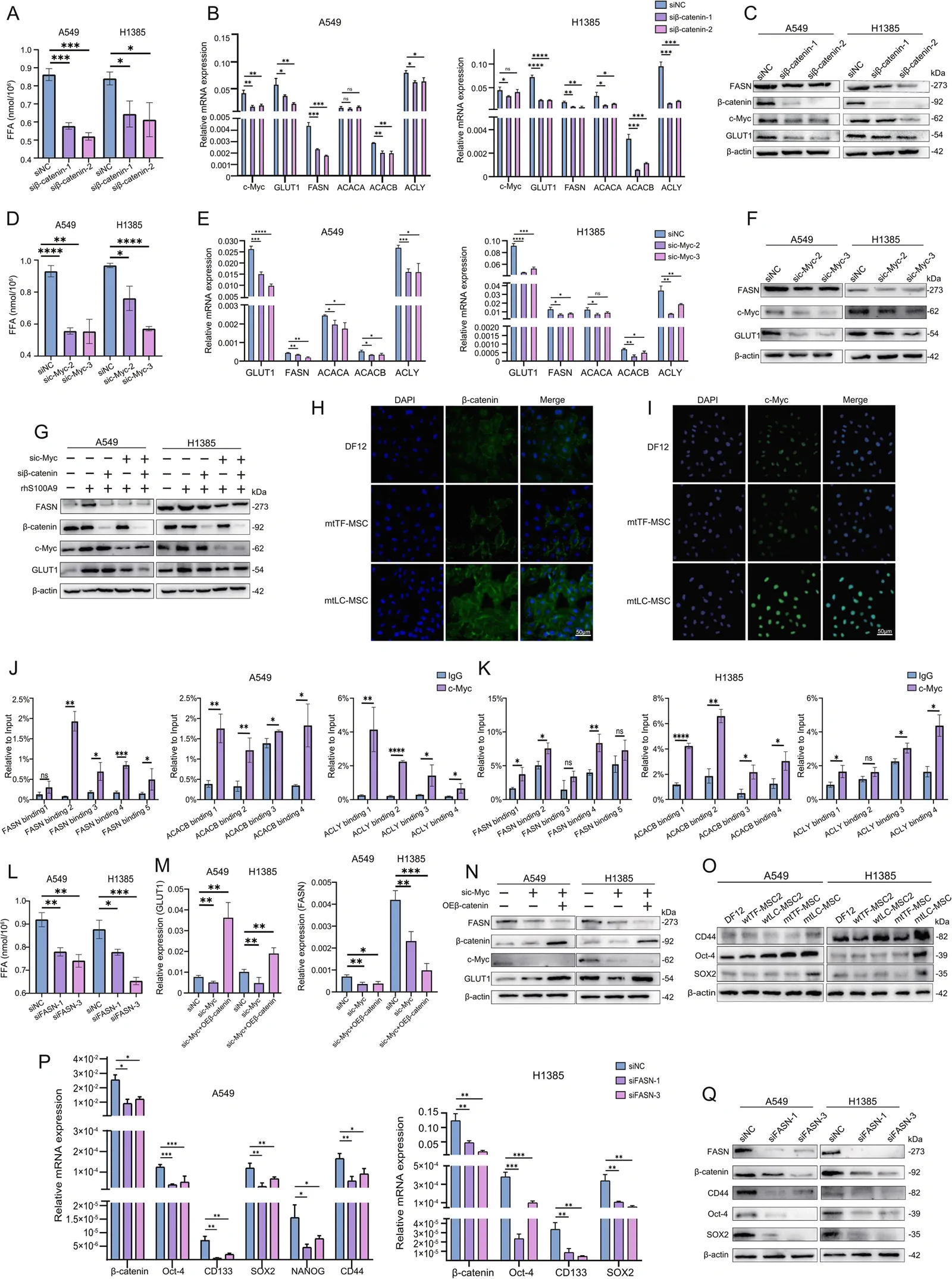
β-catenin/c-Myc axis mediates S100A9-driven fatty acid metabolic reprogramming in EGFR-wild-type tumor cells. **A** β-catenin silencing attenuated intracellular FFA levels induced by mtLC-MSCs. **B**, **C** β-catenin silencing reduced transcriptional (**B**) and protein (**C**) levels of c-Myc and downstream targets. **D** c-Myc silencing diminished intracellular FFA levels by mtLC-MSCs. **E**, **F** c-Myc silencing decreased the transcriptional (**E**) and protein (**F**) expression of downstream targets. **G** c-Myc or β-catenin silencing, alone or in combination, decreased FASN and GULT1 protein expression. **H**-**I** β-catenin translocated to cytoplasmic and nucleus (**H**), while c-Myc accumulated in nucleus (**I**) upon mtLC-MSCs-conditioned medium treatment. **J**, **K** ChIP-qPCR identified c-Myc binding sites in the promoter of downstream genes in A549 (**J**) and H1385 (**K**) cells exposed to mtLC-MSCs-conditioned medium. **L** FASN silencing reduced intracellular FFA levels upregulated by mtLC-MSCs. **M**, **N** Knockdown c-Myc concurrently overexpress β-catenin increased GLUT1, but decreased FASN expression. **O** Stem-trait protein levels under conditioned medium treatment. **P**, **Q** FASN silencing reduced the upregulated transcriptional (**P**) and protein (**Q**) levels of stem-trait proteins induced by mtLC-MSCs. NANOG and CD44 transcriptional levels were too low to be detected in H1385 cells (**P**). Consistent treatment as above described. Statistical results were shown as mean ± SD. *P < 0.05, **P < 0.01, ***P < 0.001, **** *P* < 0.0001. One-way ANOVA test except panel I (Student’s t-test). FFA, free fatty acid; GLUT1, glucose transporters 1; FASN, fatty acid synthase; ACACA, Acetyl-CoA carboxylase alpha; ACACB, Acetyl-CoA carboxylase beta; ACLY, ATP citrate lyase

We next evaluated the role of c-Myc and β-catenin in S100A9-driven lipogenesis in EGFR-wt tumor cells. Knockdown of β-catenin significantly decreased the intracellular FFA levels in A549 and H1385 cells exposed to mtLC-MSCs conditioned medium (Fig. 4A). In parallel, expression of FASN and GLUT1, the acetyl-CoA carboxylase α/β (ACACA/ACACB) and ATP citrate lyase (ACLY) were transcriptionally downregulated (Fig. 4B). FASN, GLUT1 and c-Myc protein levels were also decreased (Fig. 4C). Similarly, c-Myc knockdown led to decreased intracellular FFA levels (Fig. 4D), and reduced expression of FASN, GLUT1, ACACA, ACACB and ACLY (Fig. 4E, F). Consistent results were obtained when A549 and H1385 cells were treated with rhS100A9 and subjected to single or double knockdown of β-catenin and c-Myc (Fig. 4G; Supplementary Fig S9). 

Upon exposure to mtLC-MSCs conditioned medium, β-catenin redistributed from the inner cell membrane to the cytoplasm and subsequently translocated into the nucleus, indicating its role as a transcriptional co-activator (Fig. 4H). Meanwhile, nuclear accumulation of c-Myc was increased (Fig. 4I). Chip-qPCR further demonstrated that c-Myc binding to the FASN, ACACB and ACLY promoters was significantly enriched in both A549 and H1385 cells exposed to mtLC-MSCs conditioned medium compared with IgG controls, confirming the direct transcriptional regulation of FASN, ACACB and ACLY by c-Myc (Fig. 4J, K). Moreover, FASN knockdown itself reduced the intracellular FFA levels in A549 and H1385 cells (Fig. 4L).

Because c-Myc expression was diminished upon β-catenin knockdown (Fig. 4B, C), we next evaluated whether β-catenin regulates FASN and GLUT1 in a c-Myc-dependent manner. Overexpression of β-catenin combined with c-Myc knockdown revealed that FASN expression was reduced, whereas GLUT1 remained elevated (Fig. 4M, N). These results indicate that β-catenin regulates FASN expression through c-Myc, but controls GLUT1 expression independently. Together, our findings suggest that β-catenin and c-Myc contribute to mtLC-MSCs-induced lipogenesis, in line with activation of the citrate–acetyl-CoA–malonyl-CoA axis supporting de novo FA synthesis.

OCT4, SOX2, and β-catenin are central regulators of cancer stemness, while CD44 serves as a surface marker associated with stem-like tumor cell population [22, 23]. mtLC-MSC-conditioned medium increased β-catenin, OCT4, SOX2, and CD44 protein levels (Figs. 3C and 4O). FASN knockdown significantly diminished OCT4, SOX2, β-catenin, and CD44 at both mRNA and protein levels (Fig. 4P, Q). β-catenin and FASN formed a positive feedback loop. In addition, inhibition of FASN altered the extracellular metabolic milieu: extracellular FFA and lactate levels increased, while extracellular glucose levels decreased (Supplementary Fig S10A–C). The CD8 + and CD4 + T cells underwent robust proliferation, while their TNF-α-producing ability was significantly suppressed under mtLC-MSC-conditioned medium treatment (Supplementary Fig S10D, E). Such metabolic remodeling likely influences the interaction and functions of tumor cells, mtLC-MSCs, and immune cells within the tumor micro-regions, warranting further investigation.

Additionally, to confirm the distinction between direct and indirect reprogramming by mtLC-MSCs, we compared direct co-culture and transwell co-culture systems using equal cell number. eGFP-labeled A549 and H1385 cells were analyzed for key signaling (S100A9, c-Myc and β-catenin), metabolic (FASN and GLUT1), and stem-like regulators (CD44 and SOX2). Both systems induced these molecules to a similar extent, and free fatty acid levels were also comparable (Supplementary Fig S11). These results suggest that soluble factors contribute substantially to MSC-dependent metabolic rewiring.

### mtLC-MSCs promote EGFR-wt tumor growth and stem-like traits in vivo

**Fig. 5:**
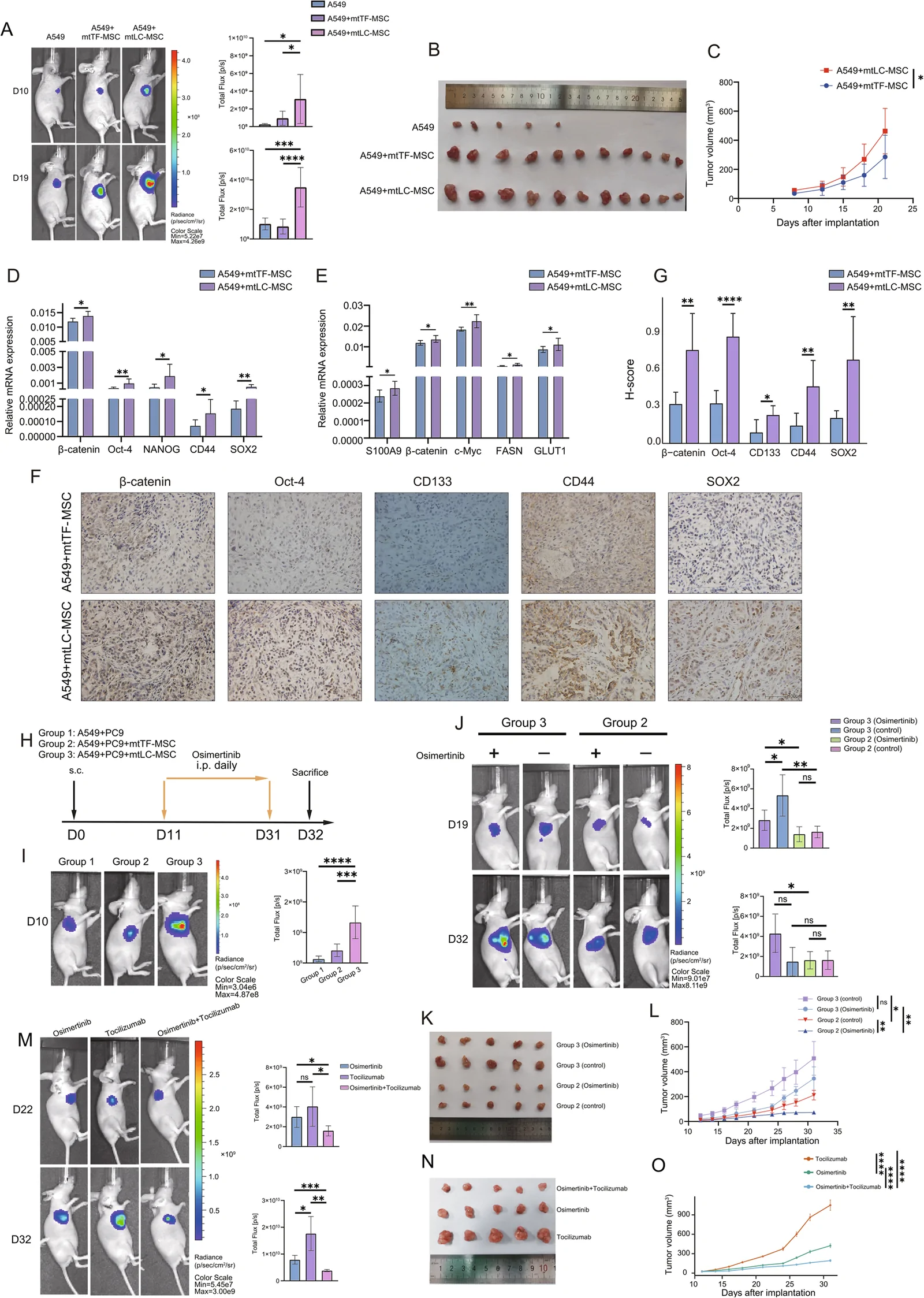
mtLC-MSCs promote EGFR-wild-type tumor growth and attenuate osimertinib efficacy in subcutaneous xenograft models. A549 cells expressed luciferase and eGFP. (**A**–**G**) A549 cells and MSCs were co-implanted. A549 alone group (n = 5); all other groups (n = 11 each). Tumor growth was monitored by live in vivo imaging (**A**); tumors were collected on day 21 when mice were sacrificed (**B**); tumor growth curve (**C**); transcriptional levels of stem-trait and S100A9 targeted genes in sored eGFP-positive A549 cells, three pooled sorted samples were prepared for each group (**D**,**E**); and stem-trait protein expression in tumors was assessed by immunohistochemical staining (**F**,**G**). (**H**–**L **) A549 cells were co-implanted with EGFR-mt PC-9 cells and mtLC/TF-MSCs to mimic EGFR-mt NSCLC. Experimental scheme, n = 10 per group before osimertinib treatment, n = 5 per group after treatment (**H**); A549 growth was assessed on day 10 (**I**); live imaging after osimertinib treatment (**J**); tumors on day 32 (**K**); and whole tumor growth curve (**L**). (**M**–**O**) Combined osimertinib with IL-6 blockade in A549/PC-9/mtLC-MSCs co-implanted model. n = 10 mice per group. Live imaging (**M**); respective tumors per group on day 32 (**N**); and whole tumor size during treatment (**O**). Osimertinib (10 mg/kg, i.p., daily). Tocilizumab (5 mg/kg, i.p., every other day). Statistical results were shown as mean ± SEM (tumor growth curves) and mean ± SD. *P < 0.05, **P < 0.01, ***P < 0.001, **** P < 0.0001. Two-way ANOVA with Dunnett’s multiple comparisons test for tumor growth curves; Student’s t-test for all others. FDR adjustment applied for multiple group comparison

Next, we investigated effect of mtLC-MSCs on A549 cell growth in vivo. A549 cells were modified to express luciferase, enabling dynamic monitoring of tumor growth and avoiding confounding measurements from co-implanted cells (Supplementary Fig S12A). In addition, stable eGFP expression in A549 and H1385 cells ensured their identification after sorting (Supplementary Fig S12B). These A549 cells were co-implanted with mtLC-MSCs or paired mtTF-MSCs isolated from the same patients in nude mice. In vivo imaging demonstrated that mtLC-MSCs significantly promoted A549 cell growth as early as 10 days after implantation, with further enhancement by day 19 (Fig. 5A), which was in line with elevated proliferation of A549 cells co-incubated with mtLC-MSCs in vitro (Supplementary Fig S13). At day 21, when mice were sacrificed, tumors in the mtLC-MSCs-co-implanted group were larger than those in the A549-alone or mtTF-MSC co-implanted group (Fig. 5B). Tumor growth curves confirmed the increased growth in the mtLC-MSCs-co-implanted group (Fig. 5C). Compared with tumor sorted from the mtTF-MSCs-co-implanted group, those from the mtLC-MSCs group displayed higher transcriptional levels of stem-associated genes (Oct-4, NANOG, CD44, SOX2) as well as S100A9, β-catenin, c-Myc, FASN, and GLUT1 (Fig. 5D, E). The elevated expression of stem-associated genes was further validated at the protein level (Fig. 5F, G). These results demonstrated that mtLC-MSCs facilitated EGFR-wt tumor cell growth in vivo. 

To characterize the growth feature of EGFR-wt tumor cells mixed with EGFR-mt tumor cells under osimertinib (TKI) treatment, EGFR-mt PC9 cells were added to the co-implantation model (Fig. 5H). Consistent with the findings in Fig. 5A, EGFR-wt A549 cells grew robustly in the presence of mtLC-MSCs, as indicated by stronger bioluminescent signals in group 3 compared with the other groups (Fig. 5I). Mice in the group 2 and 3 subsequently received daily osimertinib therapy. A549 growth was inhibited during early TKI treatment, partially reflecting the adverse environment created by PC9 cell death (Day 19, Fig. 5J). However, after a period of treatment (Day 32), group 3 still harbored the largest EGFR-wt tumors, exceeding both untreated controls and group 2 mice (Fig. 5J). These findings were corroborated by tumor size measurements (Fig. 5K, L). Meanwhile, to assess which cell population contribute to the observed growth advantages, the proportions of A549 and PC9 cells remaining in tumor tissues at the end of TKI treatment were analyzed. The results showed that EGFR-wt tumor cells preferentially persisted under TKI pressure (Supplementary Fig S14A-D), consistent with their comparative lower TKI responsiveness.

Given that IL-6 derived mtLC-MSCs was shown to promote FA remodeling and stem-like trait in EGFR-wt cells, and that IL-6 blockade reduced their growth (Supplementary Fig S15), we combined osimertinib with Tocilizumab (IL-6R antibody) in the A549/PC9/mtLC-MSCs co-implantation model. This combination not only significantly restrained A549 growth (Fig. 5M), but also synergistically suppressed overall tumor growth (Fig. 5N, O). The residual tumor cell populations were also evaluated after treatment. TKI alone resulted in persistence of A549 cells, whereas IL-6 blockade alone reduced A549 growth and allowed PC9 cells to dominate. Importantly, the combination of TKI and IL-6 blockade produced a synergistic effect, leading to markedly stronger tumor suppression of both A549 and PC9 growth, with a significant reduction in the relative proportion of PC9 cells. Nevertheless, the small residual tumors remained enriched for A549 cells, consistent with the comparatively lower TKI responsiveness of EGFR-wt cell (Supplementary Fig S16A-C). These findings highlight both the cooperative efficacy of the combination therapy and the potential role of EGFR-wt tumor cells may contribute to residual disease under TKI pressure. Collectively, these results suggest that blocking tumor-stromal interaction enhances targeted therapy efficacy in EGFR-mt NSCLC.

### mtLC-MSCs associate with EGFR-wt tumor cells in NSCLC patients

**Fig. 6:**
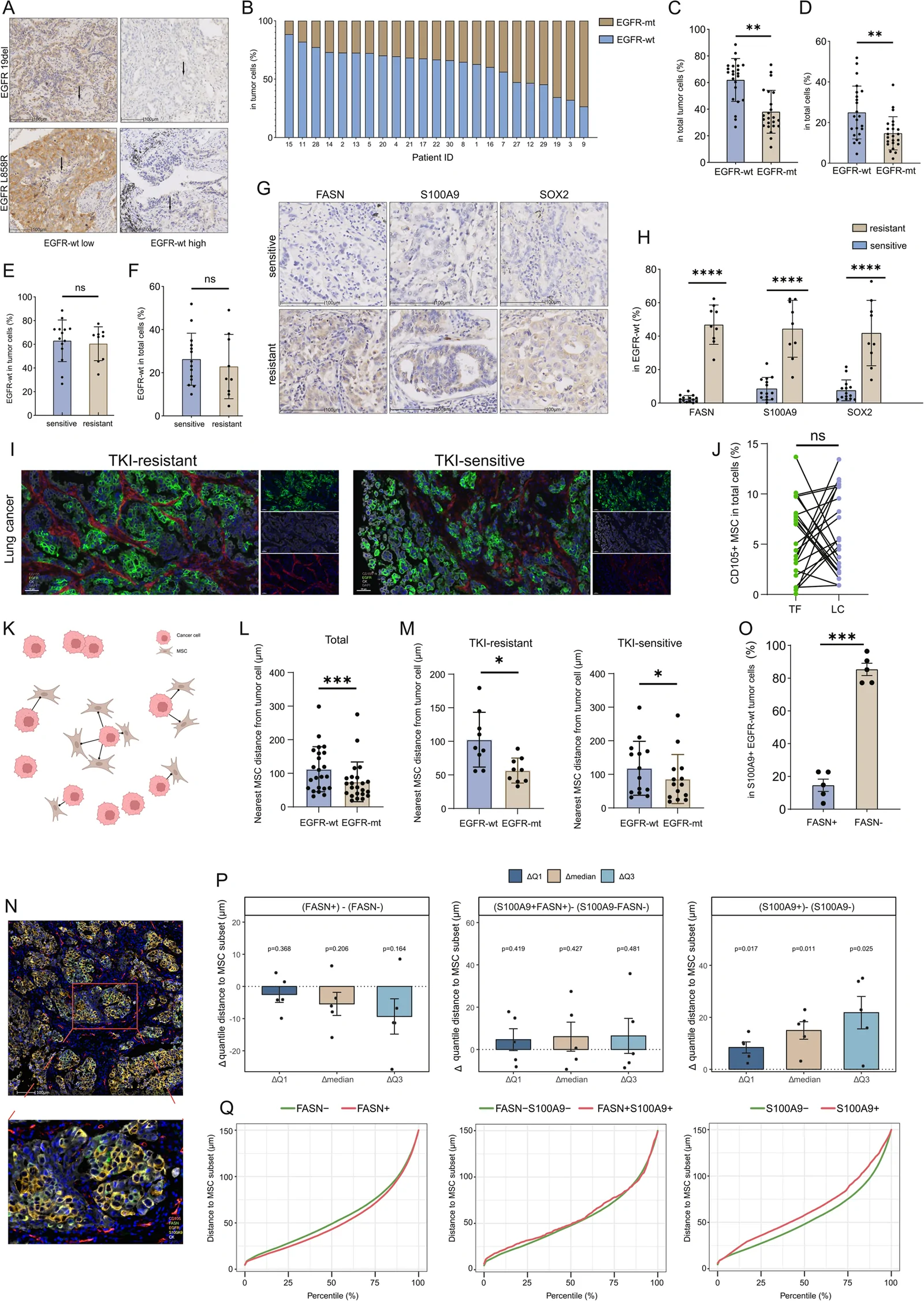
Spatial interaction of mtLC-MSC and EFGR-wt tumor cells in EGFR-mt NSCLC tissues. **A**–**H **Immunohistochemistry staining assessed EGFR-wt tumor cells. Total 23 patients with EGFR-mt NSCLC were included. Representative images showing EGFR-wt cells within mutation-specific EGFR tumor tissues (**A**); intratumoral composition of EGFR-wt and EGFR-mt tumor cells by case (**B**); paired comparison of intratumoral EGFR-wt vs. EGFR-mt (**C**) and EGFR-wt vs. EGFR-mt proportions among total cells (**D**); proportion of EGFR-wt cells in tumor cells (**E**) and in total cells (**F**) compared between TKI-sensitive and TKI-resistant groups (sensitive n=14; resistant n=9); representative images of S100A9, FASN and SOX2 expressed in TKI-sensitive and TKI-resistant tumors (**G**); and statistical these marker expression in EGFR-wt tumor cells (**H**). (**I**–**M**) Multiplex immunofluorescence staining of paired mtLC-MSCs and mtTF-MSCs (n = 23). Representative CD105+ mtLC-MSC in TKI-resistant and sensitive tumors (**I**); proportions of MSCs in paired tumor-free (TF) and lung cancer (LC) tissues (**J**); schematic diagram of spatial analysis, using each tumor as the core to calculate nearest distance to the MSCs (**K**); statistical summary of mean nearest distance, using EGFR-mt and wt tumor cells as the core (**L**); and stratification by sensitivity (**M**). **N**–**Q** Multiplex immunofluorescence staining of spatial association between mtLC-MSC subsets and EGFR-wt tumor cells (n = 5). Representative S100A9 and FASN-positive expression in mtLC-MSC (**N**); FASN expression in S100A+ mtLC-MSCs (**O**); distribution of mean nearest differences for S100A9 and FASN-positive and negative mtLC-MSC subsets shown as quantile (25th, median, 75th; ΔQ1/Δmedian/ΔQ3) (**P**); and as continuous percentile curves (**Q**). Statistical results were shown as mean ± SD except panel J, K and M (mean ± SEM). *P<0.05, **P<0.01, ***P<0.001, **** P < 0.0001. Wilcoxon signed-rank test (C, D, J, L and M), Mann–Whitney U test (E, F and H) or Student’s t-test (O and P)

Lastly, we investigated the association of S100A9-expressing mtLC-MSCs with EGFR-wt tumor cells in NSCLC patients harboring EGFR-mt tumors. Twenty-three NSCLC patients with two common EGFR mutations–EGFR 19del and EGFR L858R–who had received adjuvant TKI treatment were included in our analyses (Table 1; Fig. 6A). Tumor samples were collected at surgery. Although classified as EGFR-mt tumors, EGFR-wt tumor cells constituted a higher proportion than EGFR-mt tumor cells, both relative to total tumor cells [median: 66.78% (95% CI, 56.32–70.31) vs. 33.22% (95% CI, 27.65–39.61), *p* = 0.0039; Fig. 6B, C] and relative to total tissue cells [median: 23.07% (95% CI, 16.90–31.16) vs. 13.35% (95% CI, 9.97–16.90), *p* = 0.006; Fig. 6D]. Based on response to TKI treatment, patients were stratified into TKI-sensitive and TKI-resistant groups (14 and 9 patients, respectively). The two groups had comparable proportions of EGFR-wt tumor cells (Fig. 6E, F). However, EGFR-wt tumor cells from the resistant group exhibited significantly higher expression of FASN, S100A9, and stem-trait SOX2 compared with those from the sensitive group [median: 45.81% (95% CI, 36.80–54.92) vs. 2.12% (95% CI, 0.52–3.25), *p*<0.0001; 46.85% (95% CI, 26.11–59.78) vs. 5.90% (95% CI, 2.27–13.89), *p*<0.0001; 35.80% (95% CI, 20.89–55.24) vs. 4.93% (95% CI, 2.15–9.32), *p*<0.0001; Fig. 6G, H]. 

**Table 1:** NSCLC patient baseline characteristics

	N = 23
Sex	
Female	12 (52.2%)
Male	11 (47.8%)
Age (median; interquartile range)	
	60.0 (53.0⎼67.0)
Drinking history	
Current	5 (21.8%)
Former	1 (4.3%)
Non-drinking	17 (73.9%)
Smoking history	
Current	3 (13.0%)
Former	2 (8.7%)
Nonsmoker	18 (78.3%)
Lung lobe	
Left	6 (26.1%)
Right	17 (73.9%)
Stage	
Ⅰ	7 (30.4%)
Ⅱ	6 (26.1%)
Ⅲ	10 (43.5%)
TNM	
T T1	7 (30.4%)
T2	7 (30.4%)
T3	4 (17.4%)
T4	5 (21.8%)
N N0	14 (60.9%)
N1	3 (13.0%)
N2	6 (26.1%)
EGFR mutation types	
EGFR (E746-A750del)	14 (60.9%)
EGFR (L858R)	9 (39.1%)
Sensitivity to Adjuvant TKI Therapy	
Sensitive	14 (60.9%)
Resistant	9 (39.1%)

*Abbreviations*: *NSCLC* non–small cell lung cancer, *EGFR* epidermal growth factor receptor, *TKI *tyrosine kinase inhibitor

Using CD105 combined with spindle morphology, we quantified MSCs in paired normal and tumor lung tissues from these patients (Fig. 6I; Supplementary Fig S17A). The median proportion was 4.83% (95% CI, 4.37–7.91) in normal lung and 5.04% (95% CI, 3.83–7.21) in tumor tissues, with no statistically significant differences (*p* = 0.8229) (Fig. 6J). Specifically, within tumor tissues, whole-slide imaging analysis revealed two spatial patterns. In ~ 60% of the tumor area, mtLC-MSCs formed continuous band-like structures surrounding tumor nests. In the remaining 40% of the tumor area, mtLC-MSCs were scattered individually without forming a complete wrap. Notably, the density of mtLC-MSCs wrapping around tumor cells in the TKI-resistant samples was higher than that in the TKI-sensitive samples (Fig. 6I). TF-MSCs were diffusely distributed, with a higher density surrounding the alveolar structures (Supplementary Fig S17B).

Quantitative proximity analysis measured the shortest distance from each tumor cell to the nearest mtLC-MSCs (Fig.6K). Across all 23 patients, EGFR-mt tumor cells were significantly closer to mtLC-MSCs than EGFR-wt tumor cells (*p* = 0.0002) (Fig. 6L). Stratified by TKI response, this spatial feature held in both resistant and sensitive groups ((*p* = 0.01 and *p* = 0.01) (Fig. 6M). The preferential spatial proximity of MSCs to mutant tumor cells in both sensitive and resistant tumors suggest an intrinsic association between these cell types.

Finally, spatial interactions between mtLC-MSCs and EGFR-wt tumor cells was assessed by examining the co-expression status of S100A9 and FASN on EGFR-wt tumor cells (Fig. 6N). EGFR-wt tumor cells consistently showed higher proportions within tumor tissues compared with EGFR-mt tumor cells (Supplementary Fig S18A). S100A9 and FASN expression was observed in 7.14% (95% CI, 0–14.47) and 12.25% (95% CI, 5.73–18.77) of EGFR-wt tumor cells, respectively (Supplementary Fig S18B). And 14.82% (95% CI, 4.25–25.03) of S100A9 + EGFR-wt tumor cells co-expressed FASN (Fig. 6O). Spatial analysis revealed that EGFR-wt tumor cells with single or double-positive expression of S100A9 and FASN exhibited nearest-distance distribution to mtLC-MSCs comparable to their negative counterparts, both within the 150 μm and across the whole tumor tissue (Fig. 6P, Q; Supplementary Fig S19). Together, these findings indicated that spatial interactions between mtLC-MSCs and EGFR-wt tumor cells contribute to a heterogeneous microenvironment, which may compromise the antitumor efficacy of TKI therapy in NSCLC.

## **Discussion**

Intratumoral heterogeneity is increasingly recognized as a critical determinant of therapeutic response in EGFR-mt NSCLC [18, 24]. EGFR-wt tumor cells frequently coexist within EGFR-mt tumors, contributing to TKI resistance and relapse [7, 25]. In the study, we firstly delineate a coherent mechanism in EGFR-wt tumor cells within EGFR-mt tumors: mtLC-MSCs-derived IL-6/IL-1α induce S100A9, which then signals via TLR4/RAGE to elevate c-Myc/β-catenin. This cascade drives transcription of FASN, GLUT1, ACACA/B, ACLY, thereby promoting intracellular FFA accumulation, metabolic reprogramming, and reinforcement of cancer stem-like properties. Our findings highlight the importance of understanding the interaction between EGFR-wt tumor cells and stromal components, and provide potential targets combined with TKI to reverse resistance and improve treatment outcomes.

Paired analysis revealed that mtLC-MSCs in EGFR-mt NSCLC tumors display characteristics transcriptomic signatures, including enhanced complement, cytokine, ECM remodeling, and glycosaminoglycan biosynthesis pathways. These features indicate that MSCs under EGFR-mt TME acquire pro-inflammatory and ECM-remodifying capacities. In resistant tumors, MSCs formed denser peritumoral structures, creating biological and physical barriers that hinder immune infiltration and exacerbate tumor progression and therapeutic resistance. Besides transcriptomic profiles, conditioned media and co-culture experiments, support an important role for soluble factors and paracrine signaling in LC-MSC-mediated reprogramming. The elevated secretory function of MSC provides a plausible mechanism for regulating surrounding cells. This aligns with translational studies that engineer MSCs for secretory or exosome-based functions, suggesting that future work should explore MSC secretome profiling and functional modification. 

Our findings reveal that increased lipogenesis contributes to the acquisition of stem-like properties in EGFR-wt tumor cells residing within EGFR-mt tumors. The stemness program enables EGFR-wt cells to coexist harmoniously with their EGFR-mt counterparts, despite their intrinsically lower proliferative and cycling activity. By occupying a relatively quiescent state, EGFR-wt cells may conserve nutrients and spatial resources, indirectly supporting the growth of neighboring EGFR-mt cells. Spatial analysis showed that EGFR-mt tumor cells localized closer to mtLC-MSCs in treatment-naïve tissue, facilitating MSC education toward pro-inflammatory and ECM-remodeling phenotypes. Although TKI-treated specimens were not available, our in vivo model indicated that EGFR-mt tumor cells gained growth advantage after TKI therapy, consistent with their role in resistance. Therefore, under the selective pressure of TKI therapy, these EGFR-wt population is more likely to survive and subsequently emerge as the predominant clones in residual disease and relapse. Importantly, our in vivo data indicate that combination treatment of TKI and IL-6 signaling blockade can attenuate this adaptive lipogenic and stemness program, thereby improving therapeutic outcomes. While previously described mechanisms of TKI resistance in EGFR-mt NSCLC have focused on secondary EGFR mutations [26], bypass signaling (e.g., epithelial-mesenchymal-transition [EMT] or human epidermal growth factor receptor 2 [HER2] amplification) [27], and lineage plasticity [28], our study demonstrates a distinct contribution of EGFR-wt tumor cells through IL-6/STAT3-S100A9-driven lipogenesis and stemness. Clinically, these findings highlight the need to consider intratumoral heterogeneity and metabolic reprogramming when designing combination strategies, and they provide a rationale for integrating cytokine- or MSC-targeted therapies with TKIs to delay or overcome resistance.

Given that lipogenesis emerged as a central metabolic adaptation in EGFR-wt tumor cells, we investigated how inflammatory cues from mtLC-MSCs initiate and sustain this reprogramming. Our results illuminate that the initial drivers of this metabolic shift are inflammatory cytokines IL-6 and IL-1α secreted by mtLC-MSCs, which have been “educated” by EGFR-mt TME to adopt a pro-inflammatory phenotype. Spatial proximity between mtLC-MSCs and EGFR-wt cells provides the opportunity for paracrine communication, enabling these cytokines to activate JAK/STAT3, PI3K, and MAPK signaling and initiate metabolic reprogramming. A novel aspect of our study is the identification of the IL-6/IL-1α–S100A9–β-catenin/c-Myc axis as a central regulator of lipogenesis. S100A9-driven inflammation has been the focus of multiple studies. However, its role in linking β-catenin activation and c-Myc induction in tumors has been only rarely reported [29, 30]. Our findings provide the first evidence that S100A9, together with β-catenin and c-Myc, directly remodels metabolic gene expression in EGFR-wt tumor cells. Specifically, β-catenin regulates GLUT1 and c-Myc, c-Myc binds the promoters of FASN, ACACB, and ACLY, and both converge to drive lipogenesis, thereby establishing a mechanistic bridge between inflammatory signaling and metabolic remodeling. Our in vitro data support a major role for paracrine signaling in MSC-mediated reprogramming. These findings demonstrate the contribution of secreted molecules; however, our in vivo model with IL-6 blocked does not exclude potential roles of direct cell-cell interactions. Thus, while paracrine signaling appears to be a major mechanism, direct contact may also contribute and should be explored in future work. 

As a consequence of these signaling interactions, EGFR-wt cells exhibited a reprogrammed metabolic state in which TCA cycle activity and mitochondria oxidative phosphorylation were diverted toward anabolic lipogenesis. Inhibition of FASN not only reduced intracellular FFA but also downregulated key stemness regulators. These findings indicate that enhanced lipogenesis is tightly coupled to the maintenance of stem-like properties in EGFR-wt tumor cells. While our supplementary data suggest that intracellular metabolic rewiring may also reshape the extracellular milieu, the broader implications of these extracellular alterations warrant dedicated investigation in future studies. Taken together, these findings emphasize the metabolic reprogramming in EGFR-wt tumor cells is not a cell-autonomous event but rather the outcome of reciprocal interactions between tumor cells and TME. This metabolic crosstalk provides a survival advantage under TKI pressure and may explain the persistence of EGFR-wt clones in residual disease. 

mtLC-MSCs displayed lipid and energy metabolic similarities to BM-MSC and HUMSCS, rather than to their paired mtTF-MSCs. Although we did not trace clonal origins, prior studies suggest that tissue-resident MSCs may share development relationships with BM-derived MSCs [31]. This raises important questions about MSC ontogeny and functional diversity, warranting future investigation. Cell therapies based on HUMSCs and iPSCs-derived MSCs have been extensively investigated in regenerative medicine and cancer therapy [32, 33]. Importantly, HUMSCs did not induced teratomas in preclinical models, supporting their safety profile, whereas BM-MSCs carried tumorigenic potential. These observations highlight the need to carefully evaluate the impact of therapeutic MSCs on tumor cells and the TME when considering MSC-based interventions in cancers.

## Conclusions

Our study identifies an IL-6/IL-1α–S100A9 signaling axis that drives β-catenin/c-Myc–dependent lipogenesis in EGFR-wt tumor cells, thereby linking inflammatory cues from the tumor stroma to metabolic reprogramming and stem-like features. Importantly, we verified these mechanistic cues in patient samples, emphasizing the clinical relevance of this pathway beyond experimental models. This mechanism provides a rationale for therapeutic strategies that combine EGFR TKI with cytokine blockade or metabolic inhibition to suppress residual EGFR-wt clones and improve treatment efficacy. While additional work will be needed to define how these pathways interact with broader tumor-stroma metabolic crosstalk, our findings offer new insight into targeting lipogenesis as a strategy to enhance therapeutic outcomes in EGFR-mt NSCLC. 

## Supplementary Information

Supplementary Material 1

Supplementary Material 2

## Data Availability

The datasets generated and/or analysed during the current study are not publicly available due to privacy restrictionsbut are available from the corresponding author on reasonable request.
