# Supplementary material for: Mesenchymal stem cell-induced metabolic reprogramming of EGFR-wild-type tumor cells drives therapeutic resistance in EGFR-mutant non-small cell lung cancer
Source: J Exp Clin Cancer Res. 2026 Jul 11;45:185. doi: 10.1186/s13046-026-03748-w (PMC13499310; doi:10.1186/s13046-026-03748-w)
Supplement: Supplementary file 1 [file 13046_2026_3748_MOESM1_ESM.docx]

Supplementary Materials for

**Mesenchymal Stem Cell-induced Metabolic Reprogramming of EGFR-wild-type Tumor cells drives Therapeutic Resistance in EGFR-mutant Non-small Cell Lung Cancer**

***Additional File 1 (Word file):***

1. Materials and methods

1.1. Lentivirus transfection

1.2. Cell proliferation assay

1.3. Activation and functional evaluation in T cells

1.4. Cellular immunofluorescence

1.5. ELISA

1.6. Metabolite assays

1.7. Targeted metabolomics

1.8. Transcriptomics

2. Supplementary Figure S1–19

3. Supplementary Table S1-5

***Additional File 2 (Excel file):***

Supplementary Table S6, S7, S8, S9

1. **Materials and methods**

**1.1. Lentivirus transduction**

A549 cell lines stably expressing luciferase and enhanced green fluorescent protein (eGFP), and H1385 cell lines stably expressing eGFP, were generated by lentiviral transduction. The lentiviral particles were purchased from Shanghai Genechem Co., Ltd. (China). Cells were seeded in T-25 flasks and incubated overnight at 37 °C. The next day, the medium was replaced with the corresponding lentiviral supernatant, and the cells were transduced using HitransGP. The flasks were then centrifuged at 300 g and 25 °C for 1 hour before being returned to the incubator. After 8 hours, the medium was changed to fresh culture medium. Three days after transduction, A549 cells were selected with puromycin and hygromycin, while H1385 cells were selected with puromycin to screen for stable expression. Approximately two weeks later, stable clones were isolated and expanded. Luciferase activity and eGFP fluorescence were evaluated using a luciferase assay system and flow cytometry, respectively.

Stable luciferase-expressing A549 cells and control A549 cells were seeded in a 96-well plate at a density of 1×10⁵ and 5×10^4^ cells per well. After cell attachment, the culture medium was replaced with a pre-warmed D-luciferin working solution (0.2 mg/mL, C3654, APExBIO, USA). Following a 10-minute incubation at 37 °C, luminescence was measured and analyzed using a microplate reader.

**1.2. Cell proliferation assay**

Cell viability was assessed using the Cell Counting Kit-8 (CCK-8, HY-K0301, MCE, USA) according to the manufacturer's instructions. Briefly, cells were seeded into 96-well plates and allowed to adhere overnight. Following treatment, cells were incubated with CCK-8 reagent at a 1:10 ratio at 37°C for 1 hour before measurement. The absorbance at 450 nm was measured using a microplate reader.

**1.3. Activation and functional evaluation in T cells**

Peripheral blood mononuclear cells (PBMCs) were isolated from whole blood of five healthy donors. Briefly, PBMCs were isolated within 4 hours by density gradient centrifugation using Ficoll-Paque PLUS. PBMCs were cultured in X-VIVO 15 medium (02-060Q, Lonza, Switzerland). For T-cell activation, PBMCs at a density of 10⁶/mL were stimulated with T cell receptor signaling agonists, soluble anti-CD3 antibody OKT3 (10 ng/mL) and anti-CD28 antibody (0.1 μg/mL) for 24 hours. MSC-conditioned medium was added for additional 3 days. For flow cytometry assessment, cells were first incubated with a viability dye for 10 minutes at 4°C in the dark to discriminate live/dead cells. Subsequently, the cells were stained with fluorochrome-conjugated surface antibodies for 30 minutes at 4°C in the dark. For 2intracellular staining, cells were fixed and permeabilized using a commercial fixation/permeabilization solution (555028, BD, USA) for 30 minutes at 4°C. After two washes with permeabilization buffer, the cells were incubated with intracellular antibodies for 30 minutes at 4°C in the dark. After being washed twice, the samples were analyzed by a flow cytometer (CytoFLEX LX, Beckman, USA). To detect cytokines secreted by T cells, the cells were stimulated with a leukocyte activation cocktail with BD GolgiPlug^TM^ which was a mixture containing the phorbol ester, PMA (Phorbol 12-Myristate 13-Acetate), a calcium ionophore (Ionomycin) and the protein transport inhibitor BD GolgiPlug^TM^ (Brefeldin A) (BD, 550583, USA) for 4 hours prior to staining procedure. The flow cytometry antibodies used are listed in Supplementary Table S6.

**1.4. Cellular immunofluorescence**

Tumor cells treated with conditioned medium were seeded into 24-well plates containing glass coverslips. The cells were fixed with 4% paraformaldehyde for 15 min, washed with PBS, and permeabilized with 0.2–0.5% Triton X-100 for 10 min. After blocking with 2% BSA for 30 min and washed three times with PBS, the cells were incubated with S100A9, β-catenin and c-Myc antibodies overnight at 4 °C. Following three additional PBS washes, the cells were incubated with fluorescently labeled secondary antibodies for 1 h at room temperature in the dark. Finally, the coverslips were mounted using an anti-fade mounting medium containing DAPI, and images were captured using a fluorescence microscope.

**1.5. ELISA**

To assess protein secretion, conditioned medium from MSCs and supernatants from tumor cells exposed to conditioned medium were analyzed using specified assay kits (FineTest,

EH0201, EH0184, EH0185, China). The assay involved successive incubations with samples/biotinylated detection antibody/HRP-streptavidin, with intermittent washes. Bound enzyme activity was quantified by tetramethylbenzidine (TMB) substrate conversion, measuring the absorbance at 450 nm.

**1.6. Metabolite Assays**

Metabolite concentrations were determined using commercial assay kits according to the manufacturers' protocols. Briefly, culture supernatants were collected for L-lactate (Beyotime,

S0208S, China) and glucose (Beyotime, S0202S, China) measurements, while tumor cell samples were homogenized in assay buffer for oxaloacetate (Abcam, ab83428, USA) and citrate (Abcam, ab83396, USA) quantification. All samples were incubated with the corresponding enzyme reaction mixtures, and metabolite levels were measured spectrophotometrically or fluorometrically at specified wavelengths (570 nm for L-lactate, glucose, and oxaloacetate; Ex/Em = 535/587 nm for oxaloacetate and citrate). Standard curves constructed with provided standards were used for quantitative analysis after background correction and normalization.

**1.7. Targeted metabolomics**

All cells were treated with MSC-conditioned medium for 3 days. After PBS washing, samples from six replicate wells per group were collected, snap-frozen in liquid nitrogen, and stored at –80°C until experiments.

**1.7.1. Chromatography–mass spectrometry (GC–MS)-based quantitative analysis of medium- and long-chain fatty acids (FAs)**

(1) Metabolite extraction

Samples were thawed on ice, and a 50 µL aliquot of each was transferred to a 2-mL glass centrifuge tube. Then, 5 mL of methylene dichloride-methanol (2:1, v/v) was added. The mixture was incubated in an 80 °C water bath for 30 min to facilitate fatty acid methyl ester (FAME) derivation. After cooling, 200 µL of internal standard (IS) solution was added, followed by 1 mL of n-hexane and 5 mL of water. The mixture was vigorously vortexed, and 500 µL of the supernatant (organic layer) was collected for analysis.

(2) CM-MS analysis

FA analysis was performed using an Agilent 7890-5977 GC-MS system. Separation was achieved on an Agilent DB-23 capillary column (60 m × 250 µm × 0.15 µm). The GC oven temperature was programmed as follows: initial temperature of 50 °C (held for 3 min), ramped to 220 °C at 10 °C/min, then to 250 °C at 15 °C/min, and finally held at 250 °C for 10 min. High-purity helium was used as the carrier gas at a constant flow rate of 1.0 mL/min. The temperatures of the injection port and transfer line were set at 280 °C and 250 °C, respectively, and analysis was conducted in selected ion monitoring (SIM) mode.

(3) Data processing

Quantification of medium- and long-chain FAs was based on a calibration curve spanning a concentration range of 0.1–2500 µg/mL. The internal standard was used to normalize for injection variability and minor fluctuations in instrument response.

**1.7.2. Multiple reaction monitoring (MRM)-based quantitative analysis of energy metabolites**

(1) Metabolite extraction

Samples were retrieved from -80°C storage, and 1 mL of methanol/acetonitrile/water (2:2:1, v/v) solution was added to each sample. The mixtures were vortexed for 60 s and subjected to low-temperature ultrasonication in an ice-cold water bath for 30 min. This extraction procedure was repeated twice. Subsequently, the samples were kept at -20°C for 1 h to precipitate proteins, followed by centrifugation at 14,000 rcf for 20 min at 4°C. The resulting supernatant was collected, freeze-dried, and stored at -80°C until further analysis.

(2) CM-MS analysis

CM separation was performed using an Agilent 1290 Infinity LC UHPLC system. The mobile phase consisted of (A) 10 mM ammonium acetate in water and (B) acetonitrile. Samples were maintained at 4°C in the autosampler, and the column temperature was set to 45°C. The flow rate was 300 μL/min with an injection volume of 2 μL. The following gradient elution program was used: 0–18 min, 90% to 40% B; 18–18.1 min, 40% to 90% B; 18.1–23 min, 90% B. Quality control (QC) samples were inserted at regular intervals throughout the sample sequence to monitor system stability and reproducibility. A mixture of standard compounds related to energy metabolism was also included for retention time alignment.

MS detection was carried out on a 5500 QTRAP mass spectrometer (AB SCIEX) operated in negative ionization mode. The electrospray ionization (ESI) source parameters were set as follows: source temperature, 450°C; ion source gas 1 (Gas1), 45; ion source gas 2 (Gas2), 45; curtain gas (CUR), 30; ion spray voltage floating (ISVF), -4500 V.

(3) Data processing

Chromatographic peak areas and retention times were extracted using multiquanta software. Metabolite identification was performed by aligning retention times with authentic standards of energy metabolism-related metabolites. The concentrations of target metabolites in the samples were calculated accordingly.

**1.8. Transcriptomics**

Total RNA was extracted from cells using TRIzol reagent and qualified. mRNA was enriched using Oligo(dT)-coated magnetic beads and then fragmented by incubation with fragmentation buffer. Using the fragmented mRNA as template, first-strand cDNA was synthesized with random hexamer primers, followed by second-strand cDNA synthesis using buffer, dNTPs, and DNA polymerase I. The double-stranded cDNA was purified with AMPure XP beads. The purified cDNA was subjected to end repair, adenylation, and adapter ligation. Fragments of appropriate size were selected using AMPure XP beads, and final cDNA libraries were generated by PCR enrichment. The constructed libraries were quality-controlled by assessing insert size distribution and effective concentration. Qualified libraries were pooled according to the required sequencing depth and subjected to Illumina sequencing. Subsequent bioinformatic analysis was performed on the obtained data. DEGs were identified between compared groups. Functional enrichment analyses, including Gene Ontology (GO), Kyoto Encyclopedia of Genes and Genomes (KEGG), and Gene Set Enrichment Analysis (GSEA), were subsequently performed to interpret the biological significance of the DEGs.

2.**Supplementary Figures**

Supplementary Fig S1. Specificity of two EGFR mutation antibodies. (A) Positive staining in PC9 (exon 19 deletion) and H1975 (L858R point mutation), but negative for A549 cells (EGFR-wide-type). (B) No staining observed in normal lung epithelial cells of paired normal lung epithelium section which matched the same non-small cell lung cancer tissue section.

Supplementary Fig S2. Phenotype characterization for mtLC-MSCs (A) and mtTF-MSCs (B).

Supplementary Fig S3. IL-6 and IL-1α transcriptional expression across multiple cells. (A) mtLC-MSCs exhibited elevated transcriptional levels of IL-6. (B) EGFR-mutant H1975 tumor cells showed the highest IL-1α transcriptional expression. *P<0.05, **P<0.01, ***P<0.001, **** P < 0.0001. Student’s t-test

Supplementary Fig S4. Elevated citrate (A) and oxaloacetate (B) in H1385 and H226 cells exposed to conditioned medium.

Supplementary Fig S5. Absolute quantitative targeted metabolomics of energy metabolites and medium- and long-free fatty acids (FFAs) in A549 cells treated with conditioned medium. (A) Heatmap showed the relative levels of metabolites in A549 cells. (B) Differential bubble plot displayed the differential abundance of metabolites in A549 cells. (C) Quantification of oxaloacetate, citrate and NADPH in A549 cells. (D) Heatmap showed the relative levels of fatty acids in A549 cells. (E) Differential bubble plot displayed the differential abundance of fatty acids in A549 cells.

Supplementary Fig S6. S100A9 downstream gene–c-Myc, β-catenin, GLUT1 and FASN–transcriptional expression in EGFR-wild-type A549 and H1385 tumor cells. Tumor cells were treated with conditioned medium for 72 hours (A) or treated with recombinant human S100A9 protein for 24 hours (B).

Supplementary Fig S7. TLR4 and RAGE expression in EGFR-wild-type A549 and H1385 tumor cells.

Supplementary Fig S8. Blockade of S100A9 receptors TLR4 and RAGE attenuated transcriptional levels of downstream targets.

Supplementary Fig S9. c-Myc/β-catenin downstream genes–FASN, GLUT1, ACACA, ACACB and ACLY–transcriptional expression in EGFR-wild-type A549 and H1385 tumor cells. (A) Tumor cells were treated with mtLC-MSC-conditioned medium for 24 hour followed by Myc or β-catenin silencing, alone or in combination for 48 hours. (B) Tumor cells were treated with recombinant human S100A9 protein for 24 hours.

Supplementary Fig S10. mtLC-MSC-conditioned medium altered extracellular metabolites in EGFR-wild-type tumor cells mediated by FASN, and impact T-cell function and proliferation. (A-C) mtLC-MSC-conditioned medium treated tumor cells for 72 hours, followed by FASN silencing. (D, E) mtLC/TF-MSC- conditioned medium treated peripheral blood monocytes (PBMCs) for 72 hour, intracellular TNF-α levels (D) and ki67 expression (E) were evaluated in CD4+ and CD8+ T cells.

Supplementary Fig S11. Comparable induction of direct and indirect reprograming by mtLC-MSC. (A) Flow cytometry sorting eGFP-expressing tumor cells from mixed co-culture. Representative gating of A549-eGFP cells. (B) Protein levels of indicated regulators under direct and transwell co-culture conditions. (C) Indicated transcriptional levels of key molecules in A549 cells. (D) Free fatty acid (FFA) levels in A549 cells. Cells were cultured alone (Control), or co-cultured with mtLC-MSCs or mtTF-MSCs via Transwell (indirect) or direct contact. In direct co-culture, eGFP-labled tumor cells were flow-sorted prior to the downstream analyses.

Supplementary Fig S12. Assessment of modified A549 and H1385 cells. (A) Luciferase expression in A549-luciferase versus parental A549 cells following 10-minute incubation with 0.2 mg/mL D-luciferin. (B) eGFP levels in eGFP-expressing A549 and H1385 cells compared to their parental counterparts by flow cytometry.

Supplementary Fig 13. A549 cell proliferation detected by luciferase illuminance. A549 cells expressing luciferase were co-cultured with PC-9 and MSCs for 72 hours. 10^3^ for each subpopulation.

Supplementary Fig S14. Quantification of the relative abundance of EGFR-wild-type versus EGFR-mutant tumor cells in xenograft models. (A). Schematic representation of the in vivo experimental design. (B). Representative images of A549-eGFP (green fluorescence) and PC9 in mixed tumor xenografts. (C). Quantification of A549-eGFP+cells and PC9+ cells. (D). The ratio of A549-eGFP to PC9 in total tumor cells.

Supplementary Fig S15. Blocked of IL-6 and IL-1α pathway inhibited mtLC-MSC-conditioned medium induced cell proliferation. 103 tumor cells were pre-treated Tocilizumab or Nidanilimab, blocking IL-6 and IL-1α signaling, respectively, for one hour, followed by mtLC-MSC-conditioned medium treatment for 72 hours. CCK-8 assay.

Supplementary Fig S16. Synergistic inhibitory effects of Osimertinib and Tocilizumab on A549 and PC9 cell populations in vivo. (A). Representative immunofluorescence images of A549-eGFP cells and immunohistochemistry images of PC9 cells in tumor sections from mice treated with Osimertinib, Tocilizumab, or the combination. (B). Quantification of A549-eGFP+cells and PC9+ cells. (C). The ratio of PC9 cells to A549-eGFP cells in the indicated groups.

Supplementary Figure S17. (A). Spatial distribution of MSCs and tumor cells in TKI-resistant and TKI-sensitive lung cancer. (B). Representative images of MSC staining in paired tumor-free tissues.

Supplementary Fig S18. Multiple immunofluorescence staining of S100A9 and FASN-expressed subsets. (A) Proportions of EGFR-wt vs. EGFR-mt tumor cells among total cells. B. Subsets in EGFR-wt vs. EGFR-mt tumor cells. Data from 5 patients with EGFR-mt NSCLC.

Supplementary Fig S19. Distribution of mean nearest differences for EGFR-wt subsets shown as continuous percentile curves. Data from 5 patients with EGFR-mt NSCLC.

**Supplementary Tables**

**Table S1. siRNA oligonucleotide sequences**

| **Primer name** | **Sequence (5′→3′)** |
| --- | --- |
| siS100A9-1 Forword | CCUCCCACGAGAAGAUGCA(dT)(dT) |
| siS100A9-1 Reverse | UGCAUCUUCUCGUGGGAGG(dT)(dT) |
| siS100A9-2 Forword | GCAACAUAGAGACCAUCAU(dT)(dT) |
| siS100A9-2 Reverse | AUGAUGGUCUCUAUGUUGC(dT)(dT) |
| siS100A9-3 Forword | GCUGAGCUUCGAGGAGUUCAU(dT)(dT) |
| siS100A9-3 Reverse | AUGAACUCCUCGAAGCUCAGC(dT)(dT) |
| siβ-catenin-1 Forword | GUAAUAAGCCGGCUAUUGU(dT)(dT) |
| siβ-catenin-1 Reverse | ACAAUAGCCGGCUUAUUAC(dT)(dT) |
| siβ-catenin-2 Forword | GUUCACAACCGAAUUGUUA(dT)(dT) |
| siβ-catenin-2 Reverse | UAACAAUUCGGUUGUGAAC(dT)(dT) |
| siβ-catenin-3 Forword | CUGAAACAUGCAGUUGUAA(dT)(dT) |
| siβ-catenin-3 Reverse | UUACAACUGCAUGUUUCAG(dT)(dT) |
| sic-Myc-1 Forword | GGAACUAUGACCUCGACUA(dT)(dT) |
| sic-Myc-1 Reverse | UAGUCGAGGUCAUAGUUCC(dT)(dT) |
| sic-Myc-2 Forword | CCAAGGUAGUUAUCCUUAA(dT)(dT) |
| sic-Myc-2 Reverse | UUAAGGAUAACUACCUUGG(dT)(dT) |
| sic-Myc-3 Forword | CCUGAGACAGAUCAGCAACAA(dT)(dT) |
| sic-Myc-3 Reverse | UUGUUGCUGAUCUGUCUCAGG(dT)(dT) |
| siFASN-1 Forword | UUGGAGAGCAGCACACACC(dT)(dT) |
| siFASN-1 Reverse | GGUGUGUGCUGCUCUCCAA(dT)(dT) |
| siFASN-2 Forword | UUGUUGACAUUGUACUCGG(dT)(dT) |
| siFASN-2 Reverse | CCGAGUACAAUGUCAACAA(dT)(dT) |
| siFASN-3 Forword | UUCAGCAGGACAUUGAUGC(dT)(dT) |
| siFASN-3 Reverse | GCAUCAAUGUCCUGCUGAA(dT)(dT) |

**Table S2. Realtime-PCR primer sequences**

| **Primer name** | **Sequence (5′→3′)** | |
| --- | --- | --- |
| S100A9 Forword | ACATCATGGAGGACCTGGACAC |  |
| S100A9 Reverse | AGGTTAGCCTCGCCATCAGC |  |
| β-catenin Forword | TGGTGACAGGGAAGACATCA |  |
| β-catenin Reverse | CCATAGTGAAGGCGAACTGC |  |
| c-Myc Forword | CGTCCTCGGATTCTCTGCTCTC |  |
| c-Myc Reverse | GGTGGTGGGCGGTGTCTC |  |
| ACLY Forword | AGACCTATGACTATGCCAAGACTATCC |  |
| ACLY Reverse | GCGATGCTGCCTCCAATGATG |  |
| ACACA Forword | TCTCCTCCAACCTCAACCACTATG |  |
| ACACA Reverse | ATTCCGCCCATCCGCTGAC |  |
| ACACB Forword | AGATCGCCTCCACCGTTGTC |  |
| ACACB Reverse | TCATCTTCTGTCCACTCCACTGTC |  |
| GLUT1 Forword | TCAACCGCAACGAGGAGAACC |  |
| GLUT1 Reverse | AGCACCACAGCGATGAGGATG |  |
| FASN Forword | CAGCGGCAAGCGTGTGATG |  |
| FASN Reverse | CCTCCTCCAGCGTCCAGTTG |  |
| CD44 Forword | CCTGTTCTCTCCTGTGAAAG |  |
| CD44 Reverse | TTTGCCAATCTCTTTCATTT |  |
| NANOG Forword | GCAGTTCCAGCCAAATTCTC |  |
| NANOG Reverse | TTCACACGTCTTCAGGTTGC |  |
| SOX2 Forword | TGCGAGCGCTGCACAT |  |
| SOX2 Reverse | GCAGCGTGTACTTATCCTTCTTCA |  |
| 4-Oct Forword | GTATTCAGCCAAACGACCATC |  |
| 4-Oct Reverse | CTGGTTCGCTTTCTCTTTCG |  |
| CD133 Forword | CACCATTGACTTCTTGGTGCTG |  |
| CD133 Reverse | TGCATGCCATTTCCAAGTGG |  |
| IL1α Forword | GACCAACCAGTGCTGCTGAAGG |  |
| IL1α Reverse | GCCGTGAGTTTCCCAGAAGAAGAG |  |
| IL6 Forword | GGTGTTGCCTGCTGCCTTCC |  |
| IL6 Reverse | GTTCTGAAGAGGTGAGTGGCTGTC |  |

**Table S3. Flow cytometry antibodies**

| **Surface marker** | **Provider** | **Clone** | **catalog** | **Dilution** |
| --- | --- | --- | --- | --- |
| CD105-FITC | BioLegend | 43A3 | 323204 | 1:80 |
| CD73-APC | BioLegend | AD2 | 344006 | 1:80 |
| CD90-PE | BioLegend | 5E10 | 328110 | 1:100 |
| CD166-PC5.5 | BD Biosciences | 3A6 | 562131 | 1:40 |
| CD34-PC7 | BioLegend | 581 | 343516 | 1:40 |
| CD19-PE | BioLegend | HIB19 | 982402 | 1:40 |
| CD14-APC | BioLegend | M5E2 | 301808 | 1:40 |
| CD45-PC5.5 | BioLegend | 2D1 | 368504 | 1:100 |
| HLA-DR-FITC | BioLegend | L243 | 307604 | 1:100 |
| Mouse IgG1,κisotype Control-FITC | BD Biosciences | MOPC-21 | 555748 | 1:80 |
| Mouse IgG2a,κisotype Control-FITC | BD Biosciences | G155-178 | 555573 | 1:80 |
| Mouse IgG2a,κisotype Control-APC | BioLegend | MOPC-173 | 400222 | 1:80 |
| Mouse IgG1,κisotype Control-APC | BioLegend | MOPC-21 | 400120 | 1:80 |
| Mouse IgG1,κisotype Control-PE | BioLegend | MOPC-21 | 400113 | 1:80 |
| Mouse IgG1,κisotype Control-PC5.5 | BD Biosciences | MOPC-21 | 550795 | 1:80 |
| Mouse IgG1,κisotype Control-PC7 | BD Biosciences | MOPC-21 | 557872 | 1:80 |
| RAGE-PE | Abcam | EPR21171 | ab237363 | 1:500 |
| TLR4-APC | BioLegend | HTA | 312815 | 1:20 |
| CD4 - PC7 | BioLegend | RPA-T4 | 300512 | 1:200 |
| CD8 - PC 5.5 | BioLegend | HIT8a | 300924 | 1:20 |
| CD8 - BV510 | BD Biosciences | G42-8 | 743065 | 1:20 |
| TNF-α - PE | BioLegend | MAb11 | 502909 | 1:80 |
| Ki67 - APC | BioLegend | Ki67 | 350513 | 1:40 |
| Zombie UV | BioLegend |  | 623108 | 1:1000 |
| Cytofix/Cytoperm™ Plus  Fixation/Permeabilization Solution Kit with BD GolgiPlug™ | BD Biosciences |  | 555028 |  |
| Pharmingen™ Transcription Factor Buffer Set | BD Biosciences |  | 562574 |  |
| BD Leukocyte Activation Cocktail, with BD GolgiPlug™ | BD Biosciences |  | 550583 |  |

**Table S4. ChIP-qPCR primer names and sequences**

| **Primer name** | **Sequence (5′→3′)** |
| --- | --- |
| ACACB 1 Forword | AACCAGGCTGTCCAGGAATG |
| ACACB 1Reverse | TGAAAGGTGCACATCTCCCC |
| ACACB 2 Forword | GGGGAGATGTGCACCTTTCA |
| ACACB2 Reverse | AAAGATCAAGGGGGCAGTGG |
| ACACB3 Forword | ACTACAGACGGCTCATCCCT |
| ACACB 3 Reverse | CCACCCCTCAGCTGATGATC |
| ACACB 4 Forword | ATTGGGTCTGCACCTCCATC |
| ACACB 4 Reverse | GACGGTGGTTGCTATGGGAA |
| FASN 1 Forword | AGGTTTGGAGCATGGAGAGC |
| FASN 1 Reverse | CTCCGCGTCTTTTCCCTTCT |
| FASN 2 Forword | AGAAGGGAAAAGACGCGGAG |
| FASN 2 Reverse | CCTCCCTTTTCTGACCGCTT |
| FASN 3 Forword | CGGAGTTTCCCTGTTTCCCA |
| FASN 3 Reverse | GCTCTCCATGCTCCAAACCT |
| FASN 4 Forword | AATCAGAAGAACCCCCGCAG |
| FASN 4 Reverse | TGCGTGTCCTACTTTGAGGG |
| FASN 5 Forword | CCCTTGTCCTTCCTTGACCC |
| FASN 5 Reverse | GGCTGCGTGTCCTACTTTGA |
| ACLY1 Forword | GCAGCAGCTCACCTCAGTAA |
| ACLY1 Reverse | TTACAGGCATGTCCCACCAC |
| ACLY2 Forword | GTGGTGGGACATGCCTGTAA |
| ACLY2 Reverse | ATGGGAGGTTGCATGCAGAA |
| ACLY3 Forword | TTCTGCATGCAACCTCCCAT |
| ACLY3 Reverse | CCCTGCCCTCACCTTTTGAT |
| ACLY5 Forword | AAAGGTGCTTCGCAAGGTCT |
| ACLY5 Reverse | AACGGAGCTTCCACAAACCA |
| ACLY4 Forword | TGGTTTGTGGAAGCTCCGTT |
| ACLY4 Reverse | CGGCAGGTTGTACCCAGAAT |
| S100A9 1 Forword | ATAGAGAGAGGGGGCAGCAA |
| S100A9 1 Reverse | AGACACCCTCGGATTTGCTG |
| S100A9 2 Forword | CTCAGCCTGCTTCAACCTCA |
| S100A9 2 Reverse | TGGTAATGCTGCCAGGAAGG |
| S100A9 3 Forword | AGGATGCCAGCAAGGACTTC |
| S100A9 3 Reverse | GACTGCTCTGTGCTTCCCAT |
| S100A9 4 Forword | TAGGCAATGGGGAGCAACTG |
| S100A9 4 Reverse | CAGCAAGGGTTCCAGGAACT |

**Table S5. Clinical characteristics of patients providing tumor and adjacent normal lung for MSC isolation.**

| Patient ID | EGFR mutation type^a^ | Designation for MSCs | Gender | Age | Weight loss | Smoking | Preop KPS | Postop KPS | pTNM/Stage^b^ | Tumor location |
| --- | --- | --- | --- | --- | --- | --- | --- | --- | --- | --- |
| 1 | Exon 21 mutation | mtLC/TF-MSC | Male | 60 | No | Yes | 90 | 90 (1 month postop) | pT2bN0M0/IIA | Left Upper Lobe |
| 2 | - | wtLC/TF-MSC2 | Male | 41 | No | Yes | Not recorded | 90 (1 month postop) | pT1bN3M0/IIIB | Right Upper Lobe |
| 3 | - | wtLC/TF-MSC1 | Male | 78 | No | Yes | 90 | 90 (9 months postop) | pT2bN0M0/IIA | Right Upper Lobe |

**^a^,** assessed by routine clinical testing.

**^b^,** according to the 8th edition of the American Joint Committee on Cancer/Union for International Cancer Control (AJCC/UICC) TNM classification.

**Abbreviations:** mtLC/TF-MSC, mesenchymal stromal cells derived from EGFR-mutant lung cancer lesions or tumor-free (adjacent normal) lung tissues; wtLC/TF-MSC, mesenchymal stromal cells derived from wild-type lung cancer lesions or tumor-free (adjacent normal) lung tissues; KPS, Karnofsky Performance Status.
